# Tracing the evolving dynamics and research hotspots in the kidney neoplasm and nephron sparing surgery field from the past to the new era

**DOI:** 10.1002/cam4.7336

**Published:** 2024-06-21

**Authors:** Yuntao Yao, Yifan Liu, Tianyue Yang, Bingnan Lu, Xinyue Yang, Haoyu Zhang, Zihui Zhao, Runzhi Huang, Wang Zhou, Xiuwu Pan, Xingang Cui

**Affiliations:** ^1^ Department of Urology Xinhua Hospital Affiliated to Shanghai Jiao Tong University School of Medicine Shanghai China; ^2^ Shanghai Jiao Tong University School of Medicine Shanghai China; ^3^ Department of Burn Surgery The First Affiliated Hospital of Naval Medical University Shanghai China

**Keywords:** bibliometric analysis, ischemia, kidney neoplasm, nephron sparing surgery, partial nephrectomy, radical nephrectomy, renal cell carcinoma, renal function

## Abstract

**Background:**

With increasing detection of small renal masses and accumulating evidence that nephron sparing surgery (NSS) could achieve oncological equivalence and functional superiority compared with radical nephrectomy (RN), NSS has become first‐line therapy for some patients with localized renal masses.

**Objective:**

This study aims to review the publications in the kidney neoplasm and NSS field, exploring the research hotspots.

**Method:**

Kidney neoplasm and NSS related publications before July 3th 2023 were obtained from the Web of Science Core Collection database. We then used bibliometric analysis to conduct performance analysis, citation analysis and co‐citation network of publications, together with keyword co‐occurrence analysis.

**Results:**

Seven thousand five hundred and sixty‐four documents were finally retrieved, and the annual publications increased exponentially. The most productive authors were “KAOUK JH” and “GILL IS”, while USA, and 12 American affiliations such as CLEVELAND CLINIC FOUNDATION and MAYO CLINIC were far leading in this field. Journal of Urology and European Urology were journals with the highest citations and h‐index.

**Discussion:**

Through literature reviewing plus co‐occurrence and clustering analysis, the therapeutic effects of partial nephrectomy (PN) versus RN on patients with localized renal cell carcinoma, different operative approaches of PN, and conservative NSS methods were deemed as the most focused topics.

**Conclusion:**

Three aspects were the most important hotspots in this field. Firstly, how to provide the optimal management choices for different patients. Secondly, therapeutic effects of different management options and surgical techniques needed more prospective and randomized studies. Finally, more novel technologies and surgical techniques were required.

## Graphical abstract

Red cluster mainly discussed the therapeutic effects of PN versus RN on patients with localized RCC. In blue cluster, more conservative NSS methods including TA and AS were mainly focused. Last but not the least, we discussed different operative approaches of PN in the green cluster.

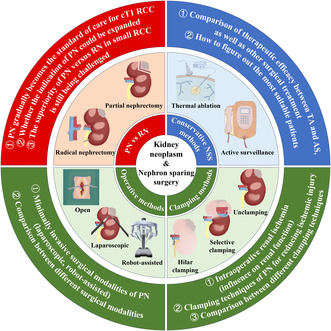

## 1 INTRODUCTION

Kidney neoplasm, includes not only the benign neoplasm (e.g., angiomyolipoma), but also the malignant renal cell carcinoma (RCC), which accounts for nearly 80% and usually requires surgical interventions and intensive therapies.^1^, ^2^, ^3^ In clinical practice, kidney neoplasm is more commonly detected by chance, due to the frequent routine use of imaging such as abdominal ultrasound.^4^, ^5^, ^6^ The growing detection of asymptomatic small renal masses (SRMs), especially clinical T1 (cT1, ≤7.0 cm) stage, has resulted in the predominant stage migration of RCC.

With increased biological and molecular understanding of RCC, surgery maintains the mainstay of curative treatment.^5^, ^6^, ^7^, ^8^, ^9^ Though radical nephrectomy (RN) was historically the golden standard of management of renal tumors, with increasing detection of small renal lesions and accumulating evidence that partial nephrectomy (PN) can achieve oncological equivalence and functional superiority, PN has been more considered as the first‐line recommendation for cT1 localized RCC.^6^, ^9^, ^10^, ^11^ Other more conservative nephron sparing surgery (NSS) approaches including thermal ablation (TA) and active surveillance (AS) have also been widely introduced into clinical practice, which avoids overtreatment, limits invasiveness and iatrogenic renal function impairment.^12^ Nowadays, there has been considerable research regarding the indication, surgical techniques, oncological and functional outcomes, for the sake of optimizing the management of patients with RCC. However, there haven't been documents that systematically investigate and summarize the development and research trends in this field.

As a result, we conducted a bibliometric analysis. Bibliometric analysis is widely conducted in literature analysis as a quantitative method for reviewing and extracting key information from the published literature.^13^ Through analyzing authors, journals, institutions, countries and keywords, the performance analysis, evolution process, and research hotspots of a field can be obtained.^14^ Bibliometrics has also been applied in research in many aspects including gut microbiota, sequencing, and various diseases,^15^, ^16^, ^17^, ^18^ which all showed great impacts. As a result, we hoped to explore the developing processes and research hotspots in the kidney neoplasm and NSS field through bibliometric analysis and literature review. In our research, we downloaded the related publications from the Web of Science core collection database to conduct the bibliometric analysis and then, based on the results and literature review, we subsequently discussed the evolving dynamics and several possible hotspots in this field.

## 2 METHOD

### 2.1 Data sources and retrieval strategies

The Web of Science (WOS) database, widely deemed as a high‐quality global literature search engine and the most appropriate database for bibliometrics analysis,^19^, ^20^, ^21^ was used for publication retrieval on 3rd July 2023. The retrieval formula was demonstrated as follows: ((TS = ((renal OR kidney) NEAR/2 (cancer* OR tumor* OR tumor* OR neoplasm* OR carcinoma* OR oncology))) AND ((TS = nephron sparing) OR (TS = partial nephrectomy))), and altogether 13,314 publications were retrieved. Next, only publication types including reviews and research articles were included, resulting in the exclusion of 3772 documents. Finally, we excluded the publications that were not in the WOS core collection database, and altogether 7564 publications were selected for the following bibliometric analysis. All the publications were downloaded into a text file, and we also uploaded them into Appendix S1.

### 2.2 Methodology of bibliometric analysis

With the help of R language (version 4.2.2, Institute for Statistics and Mathematics, Vienna, Austria; www.r‐project.org) and R studio, we used a R package called Bibliometrix (version 4.1.3) for bibliometric analysis. We put the previous text file (Appendix S1) input into Biblioshiny, which was an open‐source tool and a web‐based operator, and could provide a web operating interface for subsequent quantitative research.

### 2.3 Data analysis processes of bibliometric analysis

In our study, we conducted the performance analysis to explore the most influential authors, countries, affiliations, journals, and keywords in the area of kidney neoplasm and NSS field. We also analyzed the number of publications, citations of the publications, h‐index of authors and journals, as well as their production over time. Besides, we conducted an analysis of collaboration network, to explore the collaborative relationship between different countries. In addition, to figure out the publications that were the most influential in this area, we attached great importance to the most cited documents and references. Considering the keywords, we identified the most relevant keywords as well as trend topics over time. Then we evaluated thematic evolution, and portrayed a thematic map, hoping to catch the latest hotspots and have a general view of the development process.

In addition, arrows in the historiograph indicated that several previous influential publications were cited by the relatively newer publications, possibly demonstrating the process of development in a certain field. Furthermore, co‐citation and word co‐occurrence analyses were performed, which respectively could discover articles and words that were usually cited together by other articles, assisting us to make sense of the internal relationship and degree of betweenness about the researching hotspots of this field. The dimensionality reduction technique was utilized to better visualize the conceptual structure of keywords.

## 3 RESULTS

### 3.1 General information of our bibliometric analysis

**FIGURE 1:**
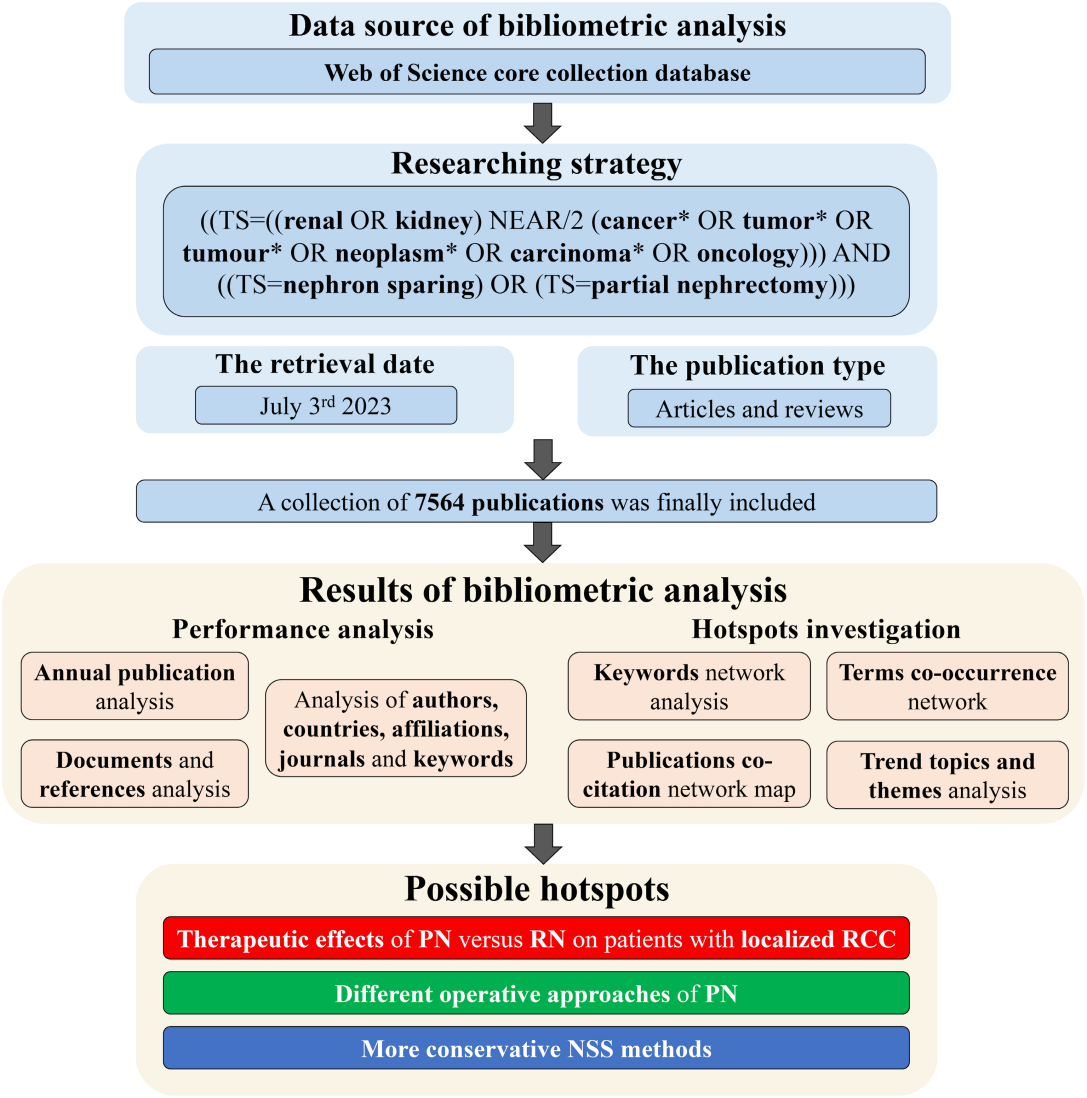
The schematic flowchart of our bibliometric analysis. We depicted the data source, processes of publications retrieval (including detailed of researching strategy, retrieval date, and publication type), and the contents of bibliometric analysis in this flowchart, and every part was distinguished by different colors. PN, partial nephrectomy; RN, radical nephrectomy; RCC, renal cell carcinoma; NSS, nephron sparing surgery.

For the sake of making our study more easily understood, we depicted a scheme and showed it in Figure 1. In total, 7564 documents were collected by us. They were authored by 23,126 individuals, and published in 769 sources (journals or books), with an average citation count of 25.85. And a total of 63,444 references were cited by these documents.

### 3.2 Analysis of annual publications

**FIGURE 2:**
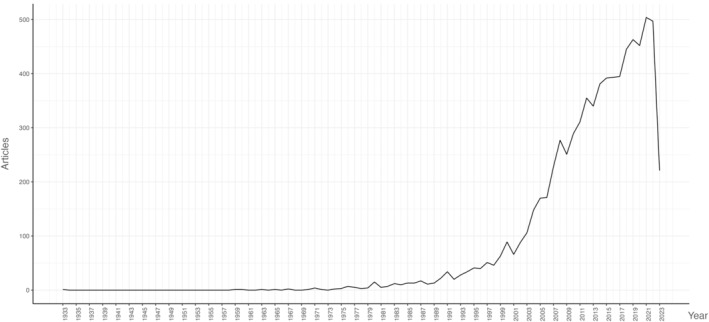
Annual publications in the kidney neoplasm and nephron sparing surgery field from 1933 to 3rd July 2023. The publications before 2000 were always less than 100, while after 2000, the yearly publications sustained exponential growth, signifying the rapid development in this field in the last two decades.

As demonstrated in Figure 2, the yearly publications sustained exponential growth after 2000, signifying the rapid development in this field in the last two decades. Although the average article citations (AACs) greatly fluctuated each year (Figure S1), it could also partly reflect the rapid development in recent two decades.

### 3.3 Contributions of authors, countries, and affiliations

The three‐field plots (Figure S2) analyzed and displayed the internal relationships based on the cocitation or co‐occurrence of terms in scientific publications. The left and right fields were key references and keywords, while the middle fields were key authors, key countries, key affiliations, and key sources, respectively, in Figure S2A–D.

The top 20 most relevant authors in the kidney neoplasm and NSS field were displayed in Table S1. “KAOUK JH” and “GILL IS” published 148 and 147 documents, respectively, surpassing other authors (Figure S3A). Besides, the total citations (TCs) of the authors were shown, with “GILL IS” and “NOVICK AC” ranking the top 2. AACs could directly reflect the average quality of the publications. The author with the highest AACs was “NOVICK AC” (132.5), and AACs of “UZZO RG” were also more than 100 (104.8). In addition, h‐index means the author has published h articles that has been cited for at least h times.^22^ It serves as a powerful index to measure the quantity and the quality of the authors' publications in the meantime, and h‐index for the top 20 authors ranged from 31 to 67. It was noteworthy “GILL IS” (67) and “NOVICK AC” (57) had the highest h‐index, and “KAOUK JH” ranked the fourth (47), suggesting their great influences in this field (Figure S3B). Moreover, local citation (LC) of a document indicated that it was cited by other documents in our retrieval set. Authors with a high number of LCs suggested their prominent impacts in this field. The most local cited authors were also “GILL IS” and “NOVICK AC”, with 8455 and 7982 LCs. Besides, other 19 authors were all cited for more than 2500 times locally (Figure S3C). The horizontal line in Figure S3D meant the timespan that an author was doing the relevant research. In addition, the bigger size and darker color shades of the nodes, respectively, represented the larger number and higher TCs per year. Nineteen of 20 authors were still actively conducting research in the recent 2 years, again suggesting this was a rapidly‐growing field. The influential publications of “KAOUK JH” and “GILL IS” mostly focused on the surgical methods including laparoscopic partial nephrectomy (LPN) and robotic‐assisted partial nephrectomy (RPN), and comparison of the therapeutic effects of RN and PN for localized renal tumors.

**FIGURE 3:**
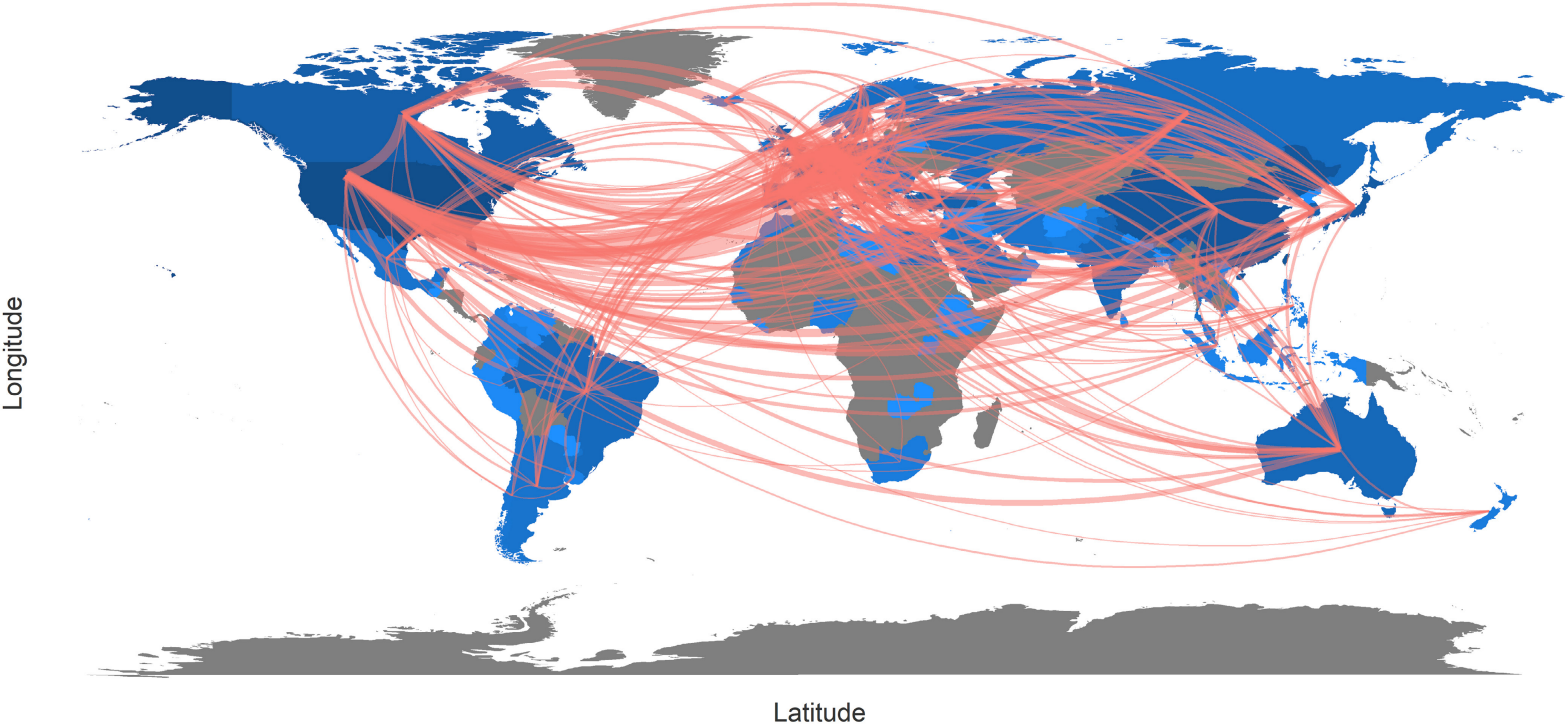
Collaboration network map between countries in the kidney neoplasm and nephron sparing surgery field. Visualizing the collaboration among countries with a network map, and the line number meant the cooperation frequencies among different countries, we could intuitively find that USA, Canada, Italy, and France were the countries more willing to cooperate. In addition, the color density was proportional to the total publications, and USA had the darkest color.

In Table S2, countries were ranked by the number of records written by corresponding authors' countries. USA was leading ahead in this field, with 2801 records (37.0%) and 113,869 citations. The following two countries were China (765 records with 6421 citations) and Japan (471 records with 5536 citations). SCP (single country publication) and MCP (multiple countries publication), respectively, means that the investigation was conducted by only one country and multiple countries. The USA, Italy, and Canada were the top three countries with most MCP (Table S2, Figure S4A), possibly suggesting that they were the most willing to cooperate, which could also be seen in the collaborative network map (Figure 3). Additionally, three clusters were identified in the country collaboration network of the top 40 productive countries (Figure S4B). USA and Canada (red), Italy and France (blue), as well as Germany (green) were the most influential and cooperative countries in the network.

The top 20 affiliations with the most publications were shown (Figure S4C). Overall, American and French affiliations were the two most preeminent affiliations in the field. Twelve American affiliations such as CLEVELAND CLINIC FOUNDATION and MAYO CLINIC, together with five French affiliations dominated the top 20 most relevant affiliations. While Figure S4D demonstrated the affiliation collaboration network, vividly showing the relationship between these affiliations.

### 3.4 Performance analysis of journals

Altogether, 769 sources (journals or books) have published articles in the kidney neoplasm and NSS field. We demonstrated the detailed information of the most relevant 20 journals, including the number of publications, impact factor (IF), and quartile in category of 2022 (Figure S5A, Table S3). These journals were all pronounced journals in the field of urology and cancer. Journal of Urology (638, 6.6, Q1), Urology (532, 2.1, Q3), Journal of Endourology (441, 2.7, Q3), BJU International (402, 4.5, Q1), European Urology (258, 23.4, Q1), Urologic Oncology‐seminars and Original Investigations (206, 2.7, Q3), and World Journal of Urology (201, 3.4, Q2) dominated the top seven accordingly. Besides, Bradford's law helped assess the exponentially diminishing returns of searching references in scientific journals. And the journals in the core sources were also the previous most relevant seven journals (Figure S5B). Among the top five most relevant journals, the number of publications of Journal of Urology has increased steadily since 1970, and all five journals sustained rapid growth after 2000 (Figure S5C). Moreover, the journals with the highest TCs and h‐index were Journal of Urology (51579, 117), European Urology (25754, 88), and Urology (17464, 65) accordingly (Table S3, Figure S5D).

### 3.5 The most influential publications

It is widely acknowledged that the number of citations for a document can not only measure its influence and importance, but also the recognition within its scientific field.^23^ The top 20 local cited documents were displayed in Table 1 and Figure S6A, and the key conclusions of each document were summarized. The publication with the highest LC (1145) proposed a nephrometry score called R.E.N.A.L to quantify and compare anatomical features of renal masses, facilitating standard characterization of renal tumor anatomical elements.^24^ The publication with second highest LC (910) was a meta‐analysis, comprehensively comparing the therapeutic efficacy of RN and various types of NSS in different population with cT1 renal mass.^25^ Besides, it was noteworthy that the document with seventh LCs (538) raised that NSS did not provide better OS compared to RN in patients with small (≤5 cm), solitary RCC, which was contradictory to many other studies.^26^ Moreover, we could find that the indication, surgical techniques, complications, oncological and functional outcomes of RN and NSS were also the major topics of other documents in Table 1,^27^, ^28^, ^29^, ^30^, ^31^, ^32^, ^33^, ^34^, ^35^, ^36^, ^37^, ^38^ which was sure to be a hotspot in this field. Besides, the European guidelines published in 2010, 2014, and 2017, which provided contemporary practical standards of care for patients, were also among the most locally cited publications.^39^, ^40^, ^41^ Figure S6B showed the 20 most totally cited documents. Even though they greatly overlapped with the most locally cited documents, there were four predominantly influential reviews published in 1996, 2009, 2016, and 2017, respectively,^5^, ^7^, ^42^, ^43^ which comprehensively discussed the various perspectives of RCC with different emphases, including developments in epidemiology, histologic classification, tumor biology, methods of diagnosis and staging, surgical techniques, and immunotherapy. Through reading these documents, we could quickly have a general view of RCC, and knew the dynamic development processes as well as the potential future directions for RCC research. Besides, Figure S6C showed the top 20 most locally cited references. The “references” referred to the references of the documents we have previously retrieved, so that references with high LCs were more likely to have great influences inside this field. For instance, the eighth most locally cited references observed graded association between chronic kidney disease (CKD) and the risk of death, cardiovascular events, and hospitalization through analyzing large, community‐based population, which was the basis of the research on the renal function (RF) after PN.^44^ Figure S6D was the references publication year spectroscopy, and the black line meant the number of cited references, while red line referred to the deviation from the 5‐year median, which reflected an overall increase in cited references in this area.

**TABLE 1:** The title, authors (year and source), key findings, and article types of the 26 publications with the highest local citations or local citations per year for kidney neoplasm and nephron sparing surgery research.

LCs (rank)	TCs (rank)	LCPY (rank)	Author (year, source)	Key conclusion	Article type
1145 (1)	1585 (2)	81.8 (2)	Kutikov A (2009, J Urology)	R.E.N.A.L. Nephrometry Score, a standardized classification system, was proposed to quantify and compare anatomical features of renal masses	Model construction
910 (2)	1455 (3)	65.0 (4)	Campbell SC (2009, J Urology)	A meta‐analysis comprehensively compared the therapeutic efficacy of RN and various types of NSS in different population with clinical T1 renal mass	Meta‐analysis (publications from January 1st, 1996 to September 30th, 2007)
813 (3)	1227 (6)	47.8 (6)	Huang WC (2006, Lancet Oncol)	RN is a significant risk factor for the development of CKD and might no longer serve as the gold standard treatment for small, renal cortical tumors (≤ 4 cm).	Retrospective cohort study (patients from 1989 to 2005)
658 (4)	1800 (1)	82.3 (1)	Ljungberg B (2015, Eur Urol)	Provides the best and most reliable evidence base for RCC management, and establishes international standards for the care of patients with RCC in 2014	Guideline
646 (5)	833 (11)	18.1 (14)	Fergany AF (2000, J Urology)	A pioneering study concludes PN is effective for localized RCC, providing long‐term tumor control with preservation of renal function	Retrospective cohort study (10‐year follow‐up)
643 (6)	911 (9)	40.2 (8)	Gill IS (2007, J Urology)	Early experience with LPN for a renal tumor ≤7 cm is promising: less operative time and blood loss, a shorter hospital stay but higher postoperative morbidity. Moreover, equivalent functional and early oncological outcomes were similar between LPN and OPN.	A comparative study (3 large referral centers, 771 patients LPN, 1029 patients OPN)
538 (7)	778 (14)	44.8 (7)	Van Poppel H (2011, Eur Urol)	A prospective and randomized study surprisingly evidenced NSS didn't provide better OS compared to RN in patients with small (≤5 cm), solitary RCC, which was contradictory to many observational studies.	A prospective RCT (541 patients with median follow‐up of 9.3 years)
535 (8)	725 (15)	24.3 (20)	Uzzo RG (2001, J Urology)	An early study raised long‐term functional advantage could be obtained through NSS with acceptable oncological outcomes.	Review
520 (9)	687 (16)	37.1 (10)	Ficarra V (2009, Eur Urol)	Put forward a simple anatomical system called PADUA that can be used as an independent predictor for the risk of surgical and medical perioperative complications in patients undergoing open NSS.	Model construction
466 (10)	652 (17)	33.3 (12)	Huang WC (2009, J Urology)	RN was associated with decreased OS and more cardiovascular events after surgery, but not significantly associated with time to first cardiovascular event or cardiovascular death. Overall, PN should more be considered in most patients with small renal tumors (≤ 4 cm).	A retrospective comparative study (2547 patients RN, 556 patients PN)
446 (11)	1122 (8)	34.3 (11)	Ljungberg B (2010, Eur Urol)	The 2010 guidelines contain information for the treatment of patients with RCC based on the current standardized general approach.	Guideline
431 (12)	579	18.7	Lau WKO (2000, Mayo Clin Proc)	A retrospective study in patients with unilateral RCC and a normal contralateral kidney suggests NSS is as effective as RN for the treatment of RCC on long‐term follow‐up. The increased risk of CKD and proteinuria after RN supports use of NSS.	A matched retrospective comparative study (164 patients in each cohort)
428 (13)	840 (10)	71.3 (3)	Campbell SC (2017, J Urology)	Several factors should be deliberated during counseling/management of patients with clinically localized renal masses, including comorbidities, oncologic and functional outcomes, and potential morbidities of various management strategies.	Guideline
392 (14)	535	26.1 (17)	Thompson RH (2008, J Urology)	Compared with PN, RN is associated with decreased OS in younger patients (< 65) with small renal masses (≤ 4 cm).	A comparative study (median follow‐up of 7.1 years, 290 patients RN, 358 patients PN)
362 (15)	531	27.8 (15)	Thompson RH (2010, Eur Urol)	Longer WIT is associated with short‐ and long‐term renal complications, suggesting every minute counts when the renal hilum is clamped. Besides, 25 min of WIT is regarded as the best cut‐off value.	A comparative study (319 patients OPN, 43 patients LPN)
360 (16)	469	18.9	Patard JJ (2004, J Urology)	Cancer specific deaths are similar between patients with renal tumors ≤7 cm in this study, suggesting that it is safe to expand the indications of PN to include patients with renal tumors up to 7 cm.	A retrospective comparative study (in 7 international centers, 1075 RN, 379 PN)
351 (17)	438	18.5	Leibovich BC (2004, J Urology)	No statistical differences exist in cancer specific survival and distant metastases‐free survival between patients treated with NSS and RN for 4–7 cm RCC in appropriately selected patients after adjusting for important clinical characteristics.	A retrospective comparative study (91 NSS, 841 RN)
339 (18)	478	14.7	Lee CT (2000, J Urology)	PN is recommended as a safe alternative for patients with a ≤ 4 cm renal tumors, due to the similar perioperative morbidity, pathological stage and outcomes after treatment with PN or RN.	A retrospective comparative study (252 patients underwent 262 procedures, 183 RN and 79 PN)
323 (19)	785 (13)	16.2	Frank I (2003, J Urology)	Increased tumor size leads to significantly higher odds of having a malignant compared to a benign tumor, clear cell compared to papillary RCC, and high grade compared to low grade malignancy.	Pathological study
317 (20)	471	15.9	Gill IS (2003, J Urology)	OPN remains the standard for NSS of renal tumors ≤7 cm, while LPN is emerging as an effective, minimally invasive therapy with longer WIT, more complications and higher rates of positive margin, but decreased narcotic use and hospital stay, as well as a more rapid convalescence.	A retrospective comparative study (200 patients)
256	807 (12)	64.0 (5)	Ljungberg B (2019, Eur Urol)	Through comprehensive and structured literature assessment with the highest methodological standards, the 2019 RCC guidelines have been updated to the most reliable contemporary evidence base for the management of RCC in 2019.	Guideline
39	144	39.0 (9)	Ljungberg B (2022, Eur Urol)	The guidelines of kidney cancer have comprehensively assessed the researches, to establish the up‐to‐date international standards for the care of patients.	Guideline (latest)
191	256	31.8 (13)	Mir MC (2017, Eur Urol)	A meta‐analysis suggested in larger localized kidney tumor (T1b and T2), PN was more recommended with acceptable cancer control, better preservation of renal function, but a higher risk of perioperative complications, which should be more carefully considered in T2 stage.	Meta‐analysis (21 case–control studies including 11,204 patients (RN 8620, PN 2584))
245	348	27.2 (16)	Scosyrev E (2014, Eur Urol)	A prospective and randomized study proved compared with RN, NSS substantially reduced the incidence of at least moderate renal dysfunction (eGFR <60), but the incidence of advanced kidney disease (eGFR <30) and kidney failure (eGFR <15) was similar. Further, the benefits of better renal function didn't increase the OS.	A prospective RCT
208	292	26.0 (18)	Thompson RH (2015, Eur Urol)	PN and percutaneous ablation for small (<7 cm) and localized renal masses are associated with similar rates of local recurrence.	A retrospective comparative study (1424 patients, 1057 PN, 180 RFA, 187 cryoablation)
51	119	25.5 (19)	Campbell SC (2021, J Urology)	Great progresses in the treatment of RCC results in the amendment. Options for intervention, including PN, RN and TA are reviewed. Careful evaluation, management, and follow‐up are emphasized.	Guideline

Abbreviations: CKD, chronic kidney disease; eGFR, estimated glomerular filtration rate; LCPY, local citations per year; LCs, local citations; LPN, laparoscopic partial nephrectomy; NSS, nephron sparing surgery; OPN, open partial nephrectomy; OS, overall survival; PN, partial nephrectomy; RCC, renal cell carcinoma, RN, radical nephrectomy; TA, thermal ablation; TCs, total citations; WIT, warm ischemia time.

Since the rank of the publications with the most LCs and TCs tended to overlook the publications that were released recently, we also displayed the top 20 documents with the most LCs per year (LCPY) in Table 1. Except for the 14 overlapped documents, the documents with the fifth and ninth LCPY were, respectively, European guidelines of 2019 and 2022, providing the newest practical standards for care of patients with kidney cancer,^6^ while the document with the nineteenth LCPY was also an amendment of the AUA guideline especially for localized cancer.^9^ Other three documents were also studies on the oncological and functional effects, plus the complications of the RN and NSS,^45^, ^46^, ^47^ further emphasizing the significance.

### 3.6 Citation analysis of the publications

Figure S7A was the historiograph and the arrows indicated the relationship of direct citation between the researchers. Except for the article published in 1999, which found that following NSS, the cancer‐free survival (CFS) is significantly better in patients with tumors ≤4 cm, so that the current TNM staging system can be improved by subdividing T1 tumors into T1a (≤4 cm) and T1b (4–7 cm),^48^ other articles have all been demonstrated in Table 1. Figure S7B was the co‐citation network, and three clusters were identified. The dominant documents in the red cluster mainly covered the following topics including guidelines, standardized classification systems for localized RCC, and factors influencing the functional outcomes of PN, such as warm ischemia time (WIT). The blue cluster mainly focused on the comparison of functional outcomes between RN and NSS, and the possible cardiovascular events after CKD. For the green clusters, it also focused on the comparison between RN and NSS, but more on the indications, surgical methods, and oncological outcomes.

### 3.7 Keywords analysis and co‐occurrence analysis

**FIGURE 4:**
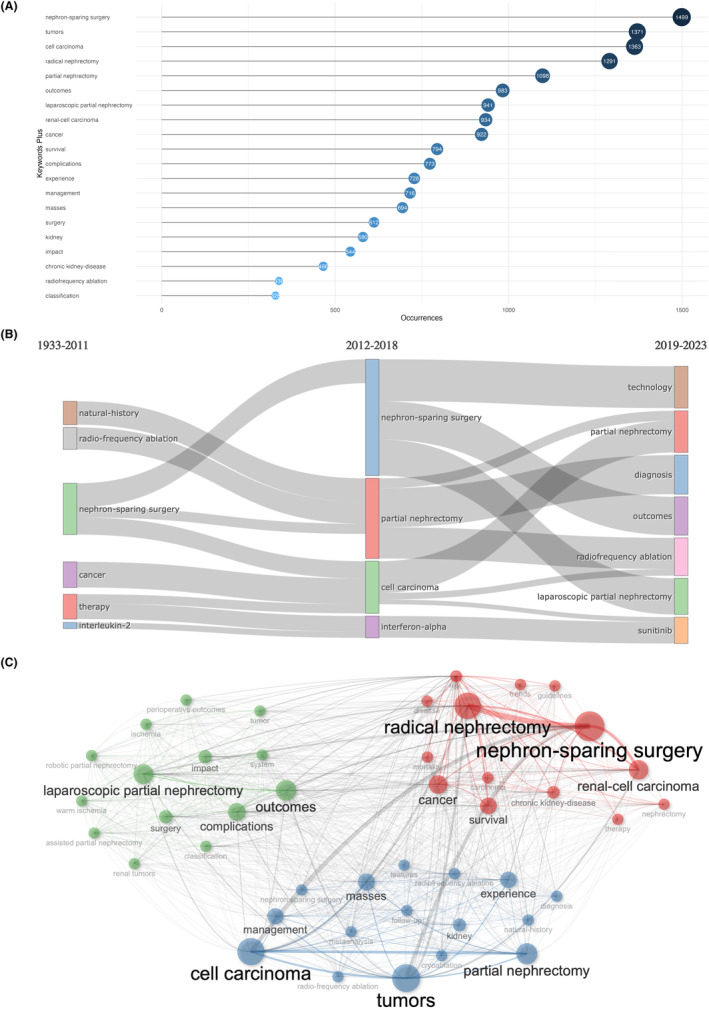
Keywords analysis and co‐occurrence analysis. (A) The top 20 most relevant keywords, mainly including different operative methods (nephron‐sparing surgery, radical nephrectomy, partial nephrectomy, laparoscopic partial nephrectomy, surgery, and radiofrequency ablation), kidney neoplasm‐related (tumors, cell carcinoma, renal‐cell carcinoma, cancer, and masses), outcomes and management of patients (outcomes, survival, complications, experience, and management), and others. (B) Evolution of keywords on the basis of occurring frequency at three different periods. Before 2011, we could find that the research focus was radio‐frequency ablation, nephron‐sparing surgery, which kept as a research hotspot in 2012–2018 and 2019–2023. In 2019–2023, with the development of robot‐assisted technology and laparoscopic partial nephrectomy, the outcomes of them were compared and assessed with the classical surgical methods. (C) Keyword co‐occurrence map shown the 40 keywords, and the three clusters were identified in red, blue, and green. The red (“radical nephrectomy” and “nephron‐sparing surgery”), blue (“cell carcinoma”, “tumors” and “partial nephrectomy”), green (“laparoscopic partial nephrectomy”, “outcomes” and “complications”) clusters were, respectively, dominated by different keywords, showing the major hotspots in this area.

The keywords of articles are highly refined, unfolding the major topics and research fields of an article together with the abstract. We extracted and analyzed keywords from the publications by bibliometric analysis to demonstrate major themes and the hotspots. The top 20 most relevant keywords were shown in Figure 4A, mainly concerning different operative methods (nephron‐sparing surgery, radical nephrectomy, partial nephrectomy, laparoscopic partial nephrectomy, surgery, and radiofrequency ablation), kidney neoplasm‐related keywords (tumors, cell carcinoma, renal‐cell carcinoma, cancer, masses, and kidney), outcomes and management of patients (outcomes, survival, complications, experience, and management), and others (impact and classification). Also, the word frequency of the top 10 keywords has all quickly growing since 2005 (Figure S8A).

The evolution of keywords on the basis of occurring frequency at three different periods (before 2011, 2012–2018, and 2019–2023) was displayed through word dynamics (Figure 4B). Before 2011, it was noticeable that the research focus was radio‐frequency ablation, nephron‐sparing surgery, which kept as a research hotspot in 2012–2018 and 2019–2023. In 2019–2023, with the development of robot‐assisted technology and laparoscopic partial nephrectomy, the outcomes of them were compared and assessed with the classical surgical methods. The development of trend topics over time since 1997 were also demonstrated in Figure S8B. Surgical methods maintained the hotspots in this field for about two decades, further confirming our foregoing conclusion that optimizing the surgical modalities was the core problem in this field. Other related topics like “trifecta outcomes”, “ischemia” of kidney, “complications”, “renal function”, “score”, and “cancer evaluation” were all key factors in determining the outcomes of nephrectomy especially PN. In addition, newly emerging topics like “perinephric fat” and “texture analysis” should also be focused on, which were crucial to the diagnosis and management of renal tumors. Figure S8C was the thematic map, which was divided into four quadrants (motor themes, niche themes, emerging or declining themes and basic and transversal themes), respectively. Two NSS methods including radio‐frequency ablation and cryoablation, together with systemic therapies including interferon‐alpha and sunitinib, were relatively more developed but less relevant to this field. While the most relevant topics further proved to be the surgical methods, as well as their influencing factors and outcomes. In Figure S9A, we presented the word cloud, showing the top 40 most frequent words, and the tree map demonstrates the top 40 most frequent words and their frequencies in the kidney neoplasm and NSS field (Figure S9B).

The keyword co‐occurrence map showed the 40 keywords, and the three clusters were identified in red, blue, and green, showing the major hotspots in this area (Figure 4C). The red cluster was dominated by “radical nephrectomy” and “nephron‐sparing surgery”, together with “renal‐cell carcinoma” and “survival”, indicating that the therapeutic effects of RN and NSS on patients with localized RCC were the most focused on. “Partial nephrectomy”, “radio‐frequency ablation”, “cytoablation”, and “tumors” were most remarkable in the blue cluster, so that the hotspots of this cluster might lie in the different NSS methods. While for the green cluster, “laparoscopic partial nephrectomy”, “outcomes”, “complications”, “robotic partial nephrectomy”, and “ischemia” were recognized, attaching great importance to different operative approaches and clamping techniques of PN.

## 4 DISCUSSION

In our study, 7564 documents related to kidney neoplasm and NSS were retrieved from the WOS core database. Increasing annual publications have manifested the popularity of the kidney neoplasm and NSS area in recent years, corresponding to its clinical significance with the increasing incidence of RCC and new surgical technologies.^4^, ^7^ “KAOUK JH”, “GILL IS”, “NOVICK AC”, and “UZZO RG” were the several most productive and influential authors in this field. For countries, USA was far leading ahead in this field. In terms of top 20 influential affiliations, 12 American affiliations together with five French affiliations dominated. Talking of the journals, Journal of Urology, European Urology, and Urology were journals with the highest TCs and h‐index. Through literature review, we found that the conclusion in this field remained controversial. Combined with the results of the co‐occurrence and clustering analysis, we wanted to discuss several possible hotspots as follows. In the red cluster, the therapeutic effects of PN versus RN on patients with localized RCC were most focused. While for the green cluster, we would discuss different operative approaches of PN. At last, more conservative NSS methods would be talked about for the green cluster.

### 4.1 The red cluster: therapeutic effects of PN versus RN on patients with localized RCC

For localized RCC, surgery remains to be the only curative treatment. While the enthusiasm in studying PN for localized renal tumors has been running high in the past decades because it ensures maximal parenchymal preservation, with satisfying oncological equivalence.^49^, ^50^ Better preserved RF could therefore reduce the risk of CKD,^27^ so that conferring a lower risk of downstream metabolic or cardiovascular sequalae, which could ultimately translate into better OS.^44^, ^51^, ^52^, ^53^ Nowadays, localized cT1 renal tumors are deemed to be best managed with PN instead of RN, regardless of the surgical approach.^6^ Then, we'd like to generally review the developing processes as well as the several current research hotspots.

#### 4.1.1 PN gradually becomes the standard of care for cT1 RCC

With RN being the standard of care for localized renal tumors for several decades since 1960s,^54^, ^55^ PN was initially more considered in patients with bilateral RCC, cancer in a solitary functioning kidney or with a contralateral kidney greatly threatened by medical diseases that might influence its future function. Under these circumstances, comparison between PN and RN revealed a similar patient outcome.^56^, ^57^ Besides, with the gradual expansion of PN into the elective setting, PN was also performed in selected patients with unilateral small localized RCC and a normal contralateral kidney, to preserve more functional renal parenchyma. An influential randomized prospective study EORTC 30904 also affirmed the oncological equivalence of PN for RCC ≤4 cm.^26^ Moreover, a lot of investigations were conducted to try to further expand the indications of PN, and noninferiority in oncological outcomes was demonstrated in both studies for patients with localized RCC ≤7 cm and 4–7 cm.^35^, ^58^, ^59^, ^60^, ^61^, ^62^, ^63^ Finally, systematic reviews have consistently shown that, for clinically localized RCC with a size of ≤7 cm, PN demonstrated superior or comparable oncological outcomes when compared to RN.^50^, ^51^, ^64^ And PN not only preserved RF and maintained a higher quality of life, but also exhibited similar rates of serious adverse events, CSS, and time to recurrence as the RN approach. Consequently, since 2014, clinical guidelines have recommended PN as the preferred management strategy for cT1 RCC.^40^

#### 4.1.2 Whether the indication of PN could be expanded remains unclear

Currently, with weak recommendation of PN for the patients with cT2 RCC and a solitary kidney or CKD in a 2022 guideline,^6^ emerging data suggested a potential role for PN in selected cases with T2 renal tumors.^47^, ^65^, ^66^, ^67^, ^68^, ^69^, ^70^, ^71^ A meta‐analysis regarded that higher surgical risk could be counterbalanced by the notable advantage of preserving more renal parenchyma for PN, with similar efficacy of PN and RN in providing oncological control.^47^ However, it also proved freedom from postoperative CKD was only significantly higher for PN in T2 patients with RENAL score ≤10. Moreover, another investigation regarding PN outcomes among the patients with larger tumors (pT2 or greater) or high‐grade tumors, found that no difference existed in cancer specific survival (CSS) compared to RN.^72^ Therefore, they drawn a conclusion that whether to perform PN or not should more depend on the technical ability to remove the tumor, instead of the stage or grade. Nevertheless, PN for the T2 renal tumors was also associated with more ischemic injury of the renal parenchyma.^25^, ^73^, ^74^, ^75^, ^76^ Besides, PN for the T2 tumors might contribute to higher risks of various complications,^47^ more positive margins,^77^, ^78^ and higher probability of incomplete tumor resection due to multi‐focality and adherent perinephric fat.^79^, ^80^ Consequently, expanding the indication of PN still needed further research, ideally in a prospective randomized way, to clarify how PN could exert its role in the demanding clinical cases with larger renal tumors.

#### 4.1.3 The superiority of PN versus RN in small localized RCC is still being challenged

Actually, debates over the therapeutic effectiveness in small localized RCC between PN and RN were still alive. Just as mentioned before, recommendation of PN for cT1 RCC stemmed from better or equivalent oncologic outcomes between RN and PN, but an increased risk of CKD and subsequent cardiovascular events for RN. However, the only prospective study (EORTC 30904) and a large retrospective study that used several propensity score techniques, did not support a survival benefit of PN versus RN.^26^, ^81^ In EORTC 30904 research, which randomized patients with renal masses of ≤5 cm and a normal contralateral kidney into PN or RN group. In the intention‐to‐treat analysis, 10 years OS was higher for RN than PN group (81.1% vs. 75.7%; HR 1.51; *p* = 0.02) despite better RF in PN group, while after adjusting pathologic features, the difference for 10 years OS was not statistically significant.^26^ Overall, the oncological equivalence and beneficial impact of PN on estimated glomerular filtration rate (eGFR) and subsequent better survival were both questioned.

Talking of the oncological equivalence, several reasons might help elucidate the controversial phenomenon in the observational studies. Firstly, selection bias possibly existed in many existing retrospective research that supports a survival advantage for PN, and more favorable renal tumors (tumor size, anatomy, and histopathology) are more likely to be surgically treated with PN. Secondly, confounding factors such as patients' general health (age and comorbidities) would probably influence the clinical decision‐making as well as outcomes of patients.^49^, ^81^, ^82^ Thirdly, the comparison of oncological effectiveness between PN and RN requires large number of patients with a long‐term follow‐up, to detect local recurrence following PN for patients with indolent tumors.^83^, ^84^, ^85^ Finally, surgical physicians performing PN might possess a higher level of expertise compared to those inclined towards RN, which also could influence the results.^82^

With regard to the functional superiority, though EORTC 30904 had notable limitations including underpowered patients and significant crossover following randomization, it still strongly suggested that PN was not superior to RN in all patients because the beneficial influence of PN on eGFR did not necessarily contribute to improved survival.^46^ In general, the CKD caused by nephrectomy might not decrease OS and preoperative eGFR also played an important role. Firstly, moderate renal dysfunction (eGFR <60 mL/min/1.73 m^2^) arising from surgery might not have the same negative implications for OS as arising from medical reasons like hypertension or diabetes mellitus (medical chronic kidney disease [CKD‐M]).^44^, ^46^, ^86^ Secondly, preoperative RF was also predominantly critical. If a patient had preoperative CKD, lower postoperative eGFR was linked to increased mortality independent of age and comorbidities.^87^ While for the patients with normal RF before surgery, the reduced nephron wouldn't be associated with worse survival unless the surgery will substantially reduce eGFR to <45 mL/min/1.73 m^2^.^88^ Besides, the perioperative complications and oncologic considerations including positive margins also influenced the therapeutic effects, and represented a key aspect in the decision‐making processes, especially for anatomically complex and large renal tumors.^49^, ^89^, ^90^, ^91^, ^92^, ^93^

Overall, current management SRMs should be individualized, with a more nuanced approach that patient factors, RF, tumor factors as well as surgical factors should all be carefully considered to make decision between PN and RN.^6^, ^9^, ^49^, ^82^, ^94^, ^95^ Consequently, considering the marked heterogeneity of the cohorts, emerging techniques such as ultrasound localization microscopy might become more important in the future.^96^, ^97^ In the future, meaningful and granular analyses that comparing the effectiveness between PN and RN are in pressing demand.

### 4.2 The green cluster: different operative approaches of PN

#### 4.2.1 Minimally invasive surgical modalities of PN

Guidelines have advocated that after selection of surgery (eg, PN vs. RN), the surgical modality should be secondly decided, highlighting its importance. During the past decades, minimally invasive surgical modalities including laparoscopic PN (LPN), robotic‐assisted PN (RPN) gradually emerged as novel and feasible alternatives to open PN (OPN).

##### Laparoscopic partial nephrectomy

LPN was an increasingly performed, minimally invasive surgical procedure in recent decades. Compared with OPN, LPN was proved to have comparable oncologic safety, faster postoperative recovery, and less morbidity with either the transperitoneal or retroperitoneal approaches.^29^, ^51^, ^98^ Interestingly, the intraoperative WIT of LPN was significantly longer than OPN, but the LPN might induce pneumoperitoneum to shield the kidney from ischemic and reperfusion injuries, and the effects remained unclear.^99^, ^100^ Nevertheless, after a matched‐pair comparison, it was found that in the immediate postoperative period, the decline in eGFR was more significant following LPN compared to OPN, while the discrepancy was no longer apparent after 3.6 years follow‐up.^101^ Moreover, LPN presented technical challenges due to the need for precise tumor margin resection and complex and time‐dependent renorrhaphy, having a steep learning curve.^102^ Overall, laparoscopic approach has been a good choice of PN for many years with some obvious benefits and drawbacks.

##### Robotic‐assisted partial nephrectomy

RPN was characterized by improved dexterity, enhanced visualization, better ergonomics, reduced surgeon physiological tremor, and decreased fatigue when compared to laparoscopic and open surgery.^103^, ^104^, ^105^ Currently, RPN has been widely used in daily clinical practices. RPN significantly outperformed OPN with fewer overall complications, fewer major complications, reduced EBL and fewer transfusion, and a shorter hospital stay in a multicenter prospective research of 1800 patients.^101^ In addition, similar results were observed regarding the WIT, operative time, complications, variation in creatinine levels, and pathological margins. However, the oncological outcomes of RPN versus OPN has only been explored in research with finite follow‐up. Furthermore, for the RPN and LPN, a comparative prospective study evaluating surgical outcomes for moderate‐to‐complex renal tumors revealed that the RPN group demonstrated significantly reduced EBL and a shorter WIT compared to the LPN group.^106^ And a systemic review and meta‐analysis also found RPN had significantly less WIT and lower complications rates than LPN.^107^ But the operative time, EBL, conversion rates, postoperative length of hospital stay, and positive margins were similar. Besides, when performed by a highly experienced robotic surgeon, RPN demonstrated a rapid learning curve to achieve WIT <20 min, console times <100 min, minimal blood loss, and satisfactory overall complication rates, which was rather technically superior to LPN.^108^ Of greater importance, rapid advances in artificial intelligence, virtual surgical models, and 5G network communication technologies had greatly enabled surgeons to perform highly demanding operations through RPN even at a long distance.^109^, ^110^, ^111^ Besides, newly‐developed laparo‐endoscopic single‐site surgery (LESS), enabled surgeons to perform laparoscopic surgery by consolidating all ports into a single incision.^112^ It was very appealing that patients undergoing LESS would have less surgical scars and comparable therapeutic effects,^113^, ^114^ and LESS was also greatly facilitated by technological expansion of robotic devices. However, a remarkable disadvantage of RPN was that RPN was more expensive than other two surgical procedures,^115^ which might restrict the usage among individuals without medical insurance and those with poorer economic conditions.

In an updated guideline, PN has been recommended to be performed using either OPN, LPN, or RPN approach, which usually accorded to the experience of surgeons and availability of equipment.^6^ Nowadays, despite limited evidence supporting the use of RPN, it has been extensively adopted in practice, especially in experienced centers. Afterwards, it is essential to conduct further comprehensive and meticulously designed research to provide more robust evidences on the patients' outcomes.

#### 4.2.2 Intraoperative renal ischemia and clamping techniques of PN

NSS frequently involves the clamping of the renal artery or arteries to interrupt renal blood flow, particularly in cases of large and anatomically complex masses with deep parenchymal invasion. The use of vascular clamping provided the surgeons with a relatively bloodless surgical field, making it easier to perform tumor resection and close the parenchymal defect. Nonetheless, the brief pause in arterial flow could result in ischemic damage to the healthy renal parenchyma, and there have been intense debates on the significance of the impact of ischemia type and duration on the long‐term RF.^116^, ^117^, ^118^ Next, we'd like to discuss the relationship between intraoperative renal ischemia, and postoperative RF after PN, as well as the clamping techniques.

##### Intraoperative renal ischemia and renal function

Histologically, it was found in the late 1970s that changes were primarily detected in the proximal tubules following WIT of 20 min, then rapidly exhibiting signs of cellular degeneration beyond 30 min, and complete cellular degeneration across all nephron levels after 60 min.^119^ And conceivably, the traditional recommendation has been to implement a maximum WIT of 30 min. In more recent studies, some investigations of renal ischemia have been conducted in the context of solitary kidneys, which was free of the compensation of the healthy contralateral kidney and regarded as a relatively ideal model.^120^, ^121^ Several observational research concluded the duration of WIT (as a continuous variable) was significantly linked to short‐term RF after renal hilar clamping.^25^, ^118^, ^122^, ^123^ Moreover, long‐term renal function could be optimized by minimizing WIT to less than 25 min.^124^ Additionally, research on renal ischemia in the context of bilateral kidneys also verified extended WIT (more than about 25–32 min) was significantly associated with loss of RF.^125^, ^126^, ^127^, ^128^, ^129^ Nevertheless, opposing views continued to persist. Findings from animal models and retrospective clinical studies indicated that WIT exceeding 30 min may still lead to complete recovery of postoperative renal function.^99^, ^130^, ^131^, ^132^, ^133^ Additionally, a prospective study demonstrated human kidneys displaying higher‐than‐expected resilience to WIT, showing minimal or no acute RF loss in the early postoperative period.^116^ And WIT even lost its significance of postoperative RF in a multivariate analysis after introducing percentage of parenchyma spared.^118^ Moreover, a review concluded that RF after PN relied most on preoperative RF and the amount of preserved renal parenchyma, rather than WIT. Besides, a recent study suggested that the histologic changes in the preserved parenchyma after clamped PN seemed to be primarily due medical comorbidities rather than ischemic injury.^134^

Though debates regarding the optimal threshold and the subsequent influences of WIT during PN still goes on, based on level 3–4 evidence from multiple studies, a time frame of 20–25 min is identified as the most precise threshold to separate patients who experience short‐ and long‐term RF decline after PN from those who don't.^25^, ^123^, ^127^, ^128^, ^135^, ^136^, ^137^

##### Various clamping techniques for reducing ischemic injury

Conceivably, it is crucial to aim for reducing ischemia time during surgical planning, particularly in cases with imperative indications,^138^ especially for LPN due to its relatively longer WIT.^119^ In response to that, several technical modifications have been proposed.^139^, ^140^ Firstly, off‐clamp PN (without clamping renal pedicle) could abolish the renal ischemia with the trade‐off of increased blood loss and a more challenging renorrhaphy.^141^ Methods including manual compression of the peri‐tumoral parenchyma and utilization of Kauffman clamps have been investigated to manage bleeding during off‐clamp PN. Overall, despite some inconsistencies, a majority of evidence indicated that the off‐clamp technique for PN, as compared to the clamped approach, resulted in a decreased risk of postoperative acute RF loss and long‐term renal impairment,^142^, ^143^, ^144^, ^145^, ^146^ especially for patients with poor preoperative RF. In addition, the off‐clamp cohort showed a slight tendency towards higher EBL, but with comparable numbers of positive margins and complication rates, indicating the potential benefits of this technique.^143^, ^145^, ^147^ Secondly, early unclamping meant clamping renal hilum solely during tumor excision and placing the initial central running suture on the renal medulla. As Gill et al reported, this technical adjustment shortened WIT for more than 50%, subsequently yielding markedly improved RF outcomes.^148^ Besides, despite early unclamping being associated with higher EBL, studies have shown that it did not impact transfusion rates or hemorrhagic complications.^149^ Thirdly, selective arterial clamping, which was guided by near‐infrared fluorescence, minimized the unnecessary renal ischemia through identifying renal vasculature and dictating the arteries that ought to be clamped.^150^, ^151^ It was firstly shown that though the patients undergoing selective arterial clamping had larger and more complex renal tumors, the EBL, complications, and postoperative RF were comparable to the clamped group.^152^ Afterwards, a larger retrospective study evidenced that patients in the selective arterial clamping group had larger and more complex tumor, but lesser RF decrease, and had a trend towards better parenchymal preservation.^153^

In addition, in cases where a longer ischemic period was expected, employing protective measures such as surface cooling to attain medullary temperatures of 15–20°C was also observed to enable a safe renal ischemia duration of 60–70 min.^154^ However, how to ideally use the cold ischemia remained a problem, and its ischemic injury to renal parenchyma was also undetermined. All these questions talked above had great clinical significances and could be the potential future research hotspots.

Nevertheless, though these techniques were established procedures that might be particularly applicable for appropriate patients with decreased baseline RF and imperative indications, these techniques were rather technically demanding with a potential for increased blood loss, and require considerable experience with PN surgery.^138^, ^155^ Consequently, these special clamping techniques ought to be performed in expert hands. More importantly, newer generation technologies like perfusion‐region three‐dimensional virtual models and new in‐house developed perfusion zone algorithm were more needed to reduce surgical difficulty.^109^, ^156^

### 4.3 The blue cluster: more conservative NSS methods

Apart from PN, there are other more conservative NSS methods available for treating clinically localized renal masses suspected to be cancer, including thermal ablation (TA) and active surveillance (AS), which has been widely used in clinical practice.

Commonly, minimally invasive thermal ablative techniques referred to cryoablation, percutaneous RFA, stereotactic radiosurgery, laser ablation, high‐intensity focused US ablation, and microwave ablation.^6^ TA is usually indicated for elderly patients with SRMs (≤4 cm lesions) who are considered unfit for surgery due to comorbidities, and patients with a genetic predisposition to develop multiple tumors, experiencing recurrences after previous surgery, or having bilateral tumors or a solitary kidney.^157^ Moreover, TA is also appropriate for individuals at high risk of experiencing complete loss of RF after undergoing PN. Regarding to the therapeutic effects, TA could provide more favorable perioperative outcomes and possible long‐term RF preservation compared with PN,^83^, ^158^ and previous research also revealed similar short‐ and medium‐term cancer‐specific results.^159^ It should be noted that this seems true even for transplant patients carrying a tumor on their kidney graft despite the few studies available.^160^ However, a contemporary meta‐analysis demonstrated that TA conferred lower local recurrence‐free survival with one treatment, while reaching therapeutic equivalences with PN only after multiple treatments.^161^, ^162^ Moreover, the therapeutic effect for cT1a patients was compared between different TA methods (heat‐based TA and cryoablation). With equivalent outcomes for the patients with tumor size ≤3 cm, cryoablation was proved to be superior in CSM for the patients with tumor size 3.1–4 cm.^163^

AS, usually involves the initial monitoring of tumor size through regular abdominal imaging, frequent check‐ups, and physical treatment. While for those who demonstrated clinical progression during follow‐up, delayed intervention was considered. Besides, before initiating surveillance, it was advised to conduct a renal biopsy.^6^ As the nonsurgical method, AS resulted in statistically significant lower CSS compared to RN and PN in patients with cT1a tumors.^68^, ^164^, ^165^ Nevertheless, in patients aged 75 years or higher, or those with a high cardiovascular risk, the advantage of surgical intervention in terms of CSS could possibly be reduced.^164^, ^165^ Moreover, several studies found that the CSS rates of AS were excellent and the metastatic rate was low within short follow up.^166^, ^167^, ^168^, ^169^, ^170^, ^171^, ^172^, ^173^, ^174^, ^175^ Consequently, in could be inferred that for the elderly and comorbid patients, especially who had incidentally detected SRMs that had relatively lower mortality compared with other competing causes, AS with delayed intervention was non‐inferior to the primary surgical interventions and could serve as a safe and cost‐effective choice for their treatment.^174^

In future, the therapeutic efficacy of these more conservative NSS methods should be more compared with other surgical treatment, to figure out the most suitable patients for these methods. Besides, the therapeutic effects among different TA methods and AS also remains elusive.

**FIGURE 5:**
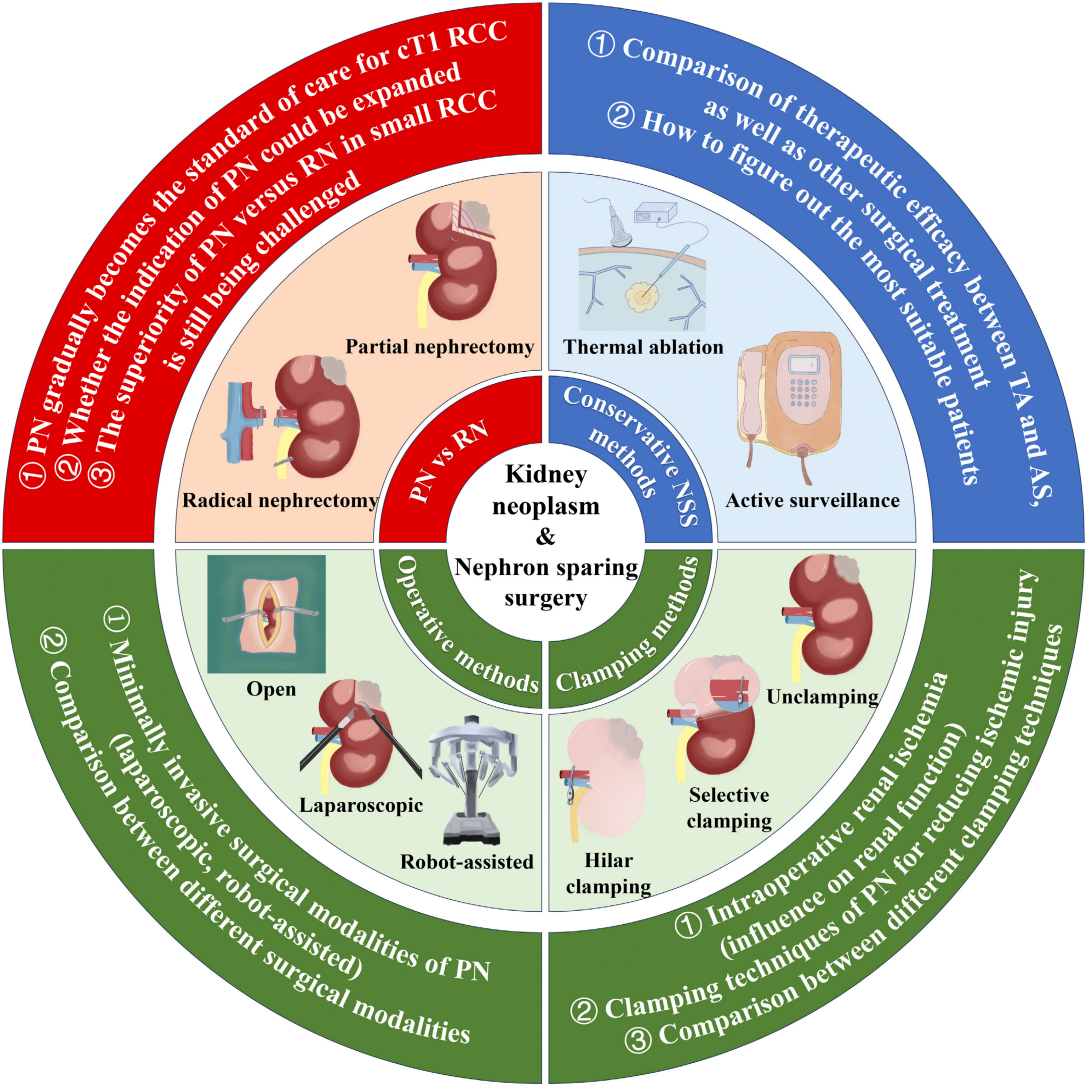
The schematic diagram vividly demonstrated the main topics in the three major clusters of our discussion section. Red cluster mainly discussed the therapeutic effects of PN versus RN on patients with localized RCC. From past to now, PN gradually becomes the standard of care for cT1 RCC, and we reviewed the general process. Then, we found that whether the indication of PN could be expanded remains unclear nowadays. Besides, the superiority of PN versus RN in small localized RCC is still being challenged by some researchers, and we summarized the perspectives of both sides. In blue cluster, more conservative NSS methods including TA and AS were mainly focused. Considering the low‐quality of the existing researches, the therapeutic efficacy of TA and AS should be more compared with each other as well as other surgical treatment. Moreover, how to figure out the most suitable patients for TA and AS was the most important issue in this field. Last but not the least, we discussed different operative approaches of PN in the green cluster. Firstly, the minimally invasive surgical modalities of PN (laparoscopic, robot‐assisted) were compared together with open PN. Secondly, we talked about the intraoperative renal ischemia (influence on renal function) and clamping techniques of PN (reduce ischemic injury). AS, active surveillance; cT1, clinical T1; NSS, nephron sparing surgery; PN, partial nephrectomy; RCC, renal cell carcinoma; RN, radical nephrectomy; TA, thermal ablation.

In the end, we summarized all the major viewpoints of our study xin Figure 5, which could intuitively demonstrate the contents discussed above.

In our study, although our paper could serve as a seminal work employing bibliometric analysis in the kidney neoplasm and NSS field, it is essential to acknowledge some inherent limitations stemming from the data acquisition process. Firstly, our retrieval only encompassed the WOS core collection database and excluded publications released after the retrieval date, potentially leading to incompleteness and outdatedness of relevant works. Secondly, even though we have analyzed the publications with the highest citations per year, some of the most recent influential studies might be overlooked. Finally, a retrieval formula is often difficult to balance between comprehensive and precise searches, inevitably leading to either an expanded search scope or the omission of some relevant literature that should have been included in the search.

## 5 CONCLUSION

The bibliometric analysis revealed research on kidney neoplasm and NSS were growing. Three aspects were found to be the most important hotspots currently. Firstly, though most patients in cT1 stage could benefit more when receiving PN, how to provide the optimal management choices for patients with different characteristics remains elusive and is now largely based surgical experience. Secondly, the therapeutic effects of oncological and functional outcomes of different management options and surgical techniques needed more prospective and randomized studies to provide robust evidences. Finally, more novel technologies and surgical techniques were required to improve therapeutic efficacy, to make challenging surgeries easier, and to further promote telesurgery.

## AUTHOR CONTRIBUTIONS

**Yuntao Yao:** Conceptualization (lead); data curation (equal); formal analysis (equal); investigation (equal); methodology (equal); project administration (equal); resources (equal); software (equal); supervision (equal); validation (equal); visualization (equal); writing – original draft (lead). **Yifan Liu:** Conceptualization (equal); data curation (equal); formal analysis (equal); investigation (equal); methodology (equal); project administration (equal); resources (equal); software (equal); supervision (equal); validation (equal); visualization (equal); writing – original draft (equal). **Tianyue Yang:** Conceptualization (equal); data curation (equal); formal analysis (equal); investigation (equal); methodology (equal); project administration (equal); resources (equal); software (equal); supervision (equal); validation (equal); visualization (lead); writing – original draft (equal). **Bingnan Lu:** Conceptualization (equal); data curation (equal); formal analysis (equal); investigation (equal); methodology (equal); project administration (equal); resources (equal); software (equal); supervision (equal); validation (equal); visualization (equal); writing – original draft (equal). **Xinyue Yang:** Conceptualization (equal); data curation (equal); formal analysis (equal); investigation (equal); methodology (equal); project administration (equal); resources (equal); software (equal); supervision (equal); validation (equal); visualization (equal); writing – original draft (supporting). **Haoyu Zhang:** Conceptualization (equal); data curation (equal); formal analysis (equal); investigation (equal); methodology (equal); project administration (equal); resources (equal); software (equal); supervision (equal); validation (equal); visualization (equal); writing – original draft (supporting). **Zihui Zhao:** Conceptualization (equal); data curation (equal); formal analysis (equal); investigation (equal); methodology (equal); project administration (equal); resources (equal); software (equal); supervision (equal); validation (equal); visualization (equal); writing – original draft (supporting). **Runzhi Huang:** Conceptualization (equal); writing – review and editing (equal). **Wang Zhou:** Conceptualization (equal); writing – review and editing (equal). **Xiuwu Pan:** Conceptualization (equal); writing – review and editing (equal). **Xingang Cui:** Funding acquisition (lead); writing – review and editing (lead).

## FUNDING INFORMATION

This work was sponsored by the National Natural Science Foundation of China (No. 81974391, 82072806, 82173265); Leading health talents of Shanghai Municipal Health Commission (2022LJ002); Shanghai Rising‐Star Program (23QC1401400); the Natural Science Foundation of Shanghai (23ZR1441300); Shanghai Municipal Commission of Health and Family Planning (20204Y0042); Hospital Funded Clinical Research, Xinhua Hospital Affiliated to Shanghai Jiao Tong University School of Medicine (21XHDB06).

## CONFLICT OF INTEREST STATEMENT

The authors declare that there is no conflict of interests.

## ETHICS STATEMENT

The study was approved by the Ethics Committee of Xinhua Hospital Affiliated to Shanghai Jiao Tong University School of Medicine. All authors confirmed that all methods were carried out in accordance with relevant guidelines and regulations.

## Supporting information


Appendix S1.

## Data Availability

The datasets generated and/or analyzed during the current study are available on the Web of Science™ (WOS), and the datasets generated and/or analysed during the current study are available in the supplementary material.
