# Supplementary material for: Tracing the evolving dynamics and research hotspots in the kidney neoplasm and nephron sparing surgery field from the past to the new era
Source: Cancer Med. 2024 Jun 21;13(12):e7336. doi: 10.1002/cam4.7336 (PMC11192648; doi:10.1002/cam4.7336)
Supplement: Supplementary file 1 — Appendix S1. [file CAM4-13-e7336-s001.docx]

**Supplementary Figures**

**
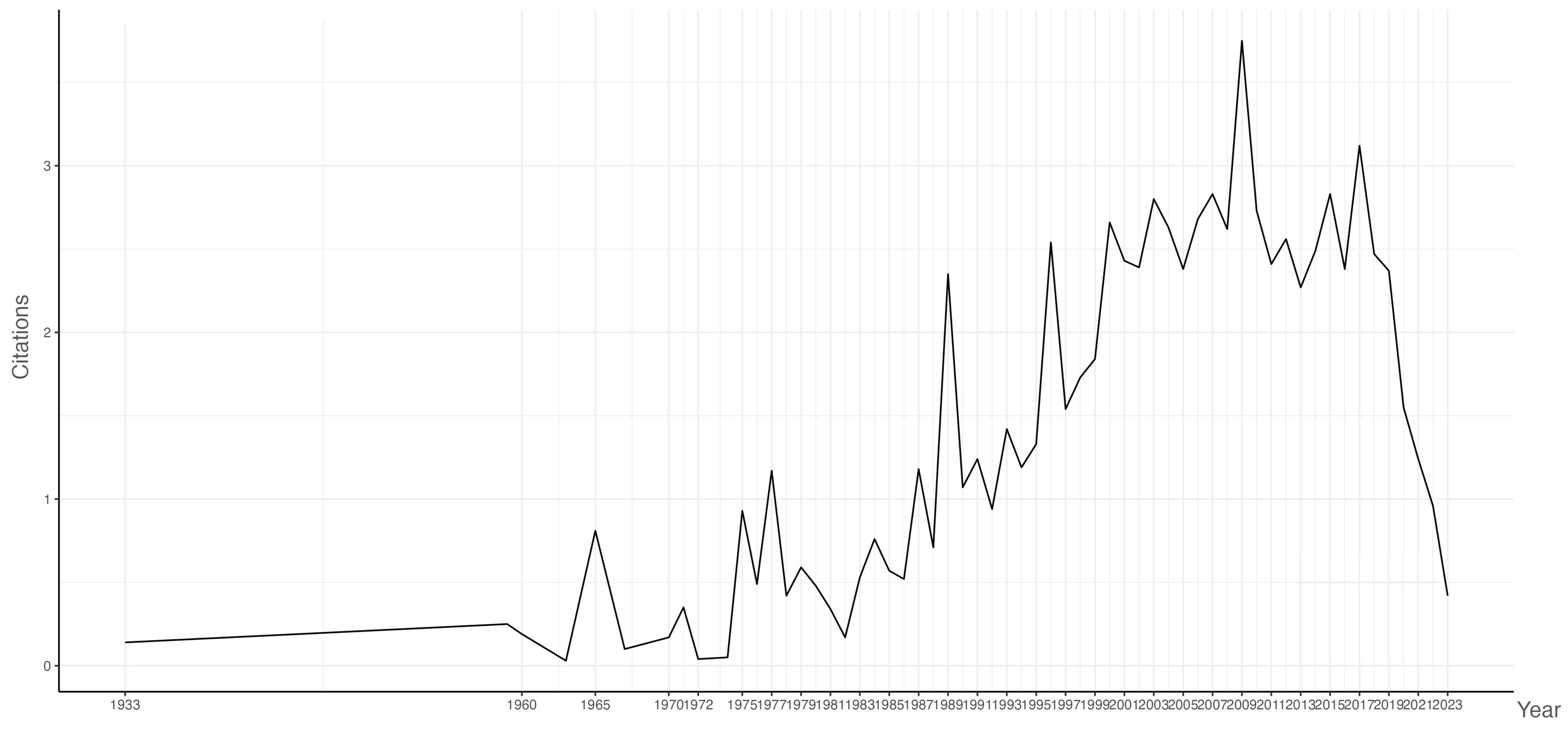
**

**Figure S1** | Average citations per year.

The average citations of the publications in the kidney neoplasm and nephron sparing surgery field demonstrated fluctuations in different years, and the average citations in recent two decades were relatively higher, with about 2-3 citations per article.

**
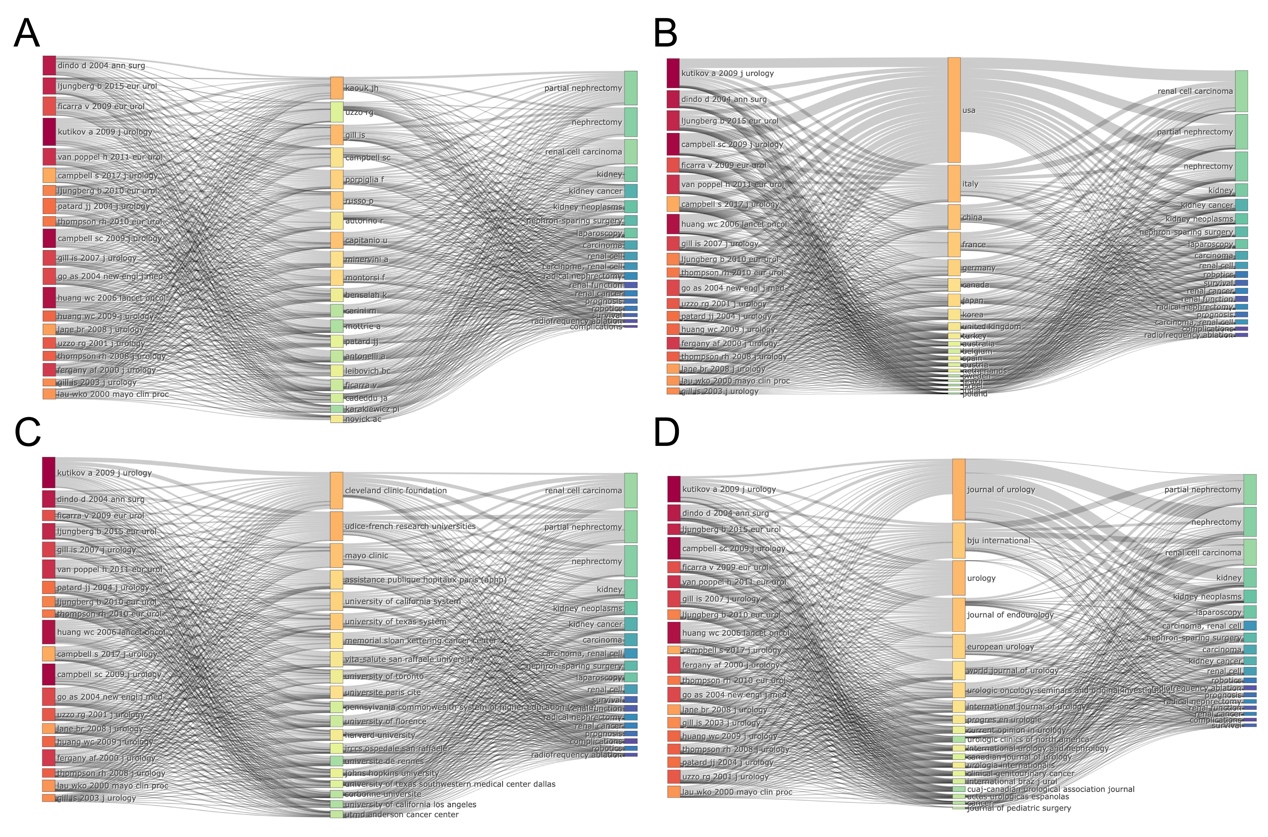
**

**Figure S2** | Overview of the kidney neoplasm and nephron sparing surgery field by three-field plots.

**(A)** Based on co-citation or co-occurrence of terms in scientific publications of this field, the three-field plots analyzed and displayed the relationships among key references, key authors and keywords from left to right respectively.

**(B)** Left field, middle field, and right field represent key references, key countries and keywords respectively.

**(C)** Left field, middle field, and right field represent key references, key affiliations and keywords respectively.

**(D)** Left field, middle field, and right field represent key references, key sources and keywords respectively.

| **Rank** | **Authors** | **Publications** | **TCs** | **AACs** | **H-index** | **LCs** |
| --- | --- | --- | --- | --- | --- | --- |
| **1** | KAOUK JH | 148 | 10340 | 69.9 | 47 | 6701 |
| **2** | GILL IS | 147 | 13397 | 91.1 | 67 | 8455 |
| **3** | CAPITANIO U | 133 | 3535 | 26.6 | 31 | 1855 |
| **4** | RUSSO P | 129 | 10230 | 79.3 | 51 | 5450 |
| **5** | PORPIGLIA F | 128 | 4095 | 32.0 | 38 | 2838 |
| **6** | MONTORSI F | 114 | 4498 | 39.5 | 35 | 2480 |
| **7** | MINERVINI A | 111 | 2869 | 25.8 | 30 | 2070 |
| **8** | CAMPBELL SC | 108 | 8835 | 81.8 | 41 | 4960 |
| **9** | AUTORINO R | 99 | 3105 | 31.4 | 32 | 2044 |
| **10** | NOVICK AC | 98 | 12985 | 132.5 | 57 | 7982 |
| **11** | BENSALAH K | 95 | 3990 | 42.0 | 27 | 2001 |
| **12** | LEIBOVICH BC | 92 | 7562 | 82.2 | 43 | 3975 |
| **13** | PATARD JJ | 91 | 6045 | 66.4 | 39 | 3013 |
| **14** | UZZO RG | 89 | 9331 | 104.8 | 38 | 5576 |
| **15** | CADEDDU JA | 87 | 4057 | 46.6 | 30 | 2200 |
| **16** | FICARRA V | 85 | 6605 | 77.7 | 41 | 2986 |
| **17** | ANTONELLI A | 81 | 2151 | 26.6 | 25 | 1530 |
| **18** | CARINI M | 81 | 2782 | 34.3 | 29 | 2055 |
| **19** | MOTTRIE A | 81 | 2155 | 26.6 | 26 | 1495 |
| **20** | KARAKIEWICZ PI | 79 | 2913 | 36.9 | 30 | 1527 |

**Table S1 |** Top 20 most contributing authors in the kidney neoplasm and nephron sparing surgery field. Abbreviations: TCs, total citations; AACs, average article citations; LCs, local citations;

**
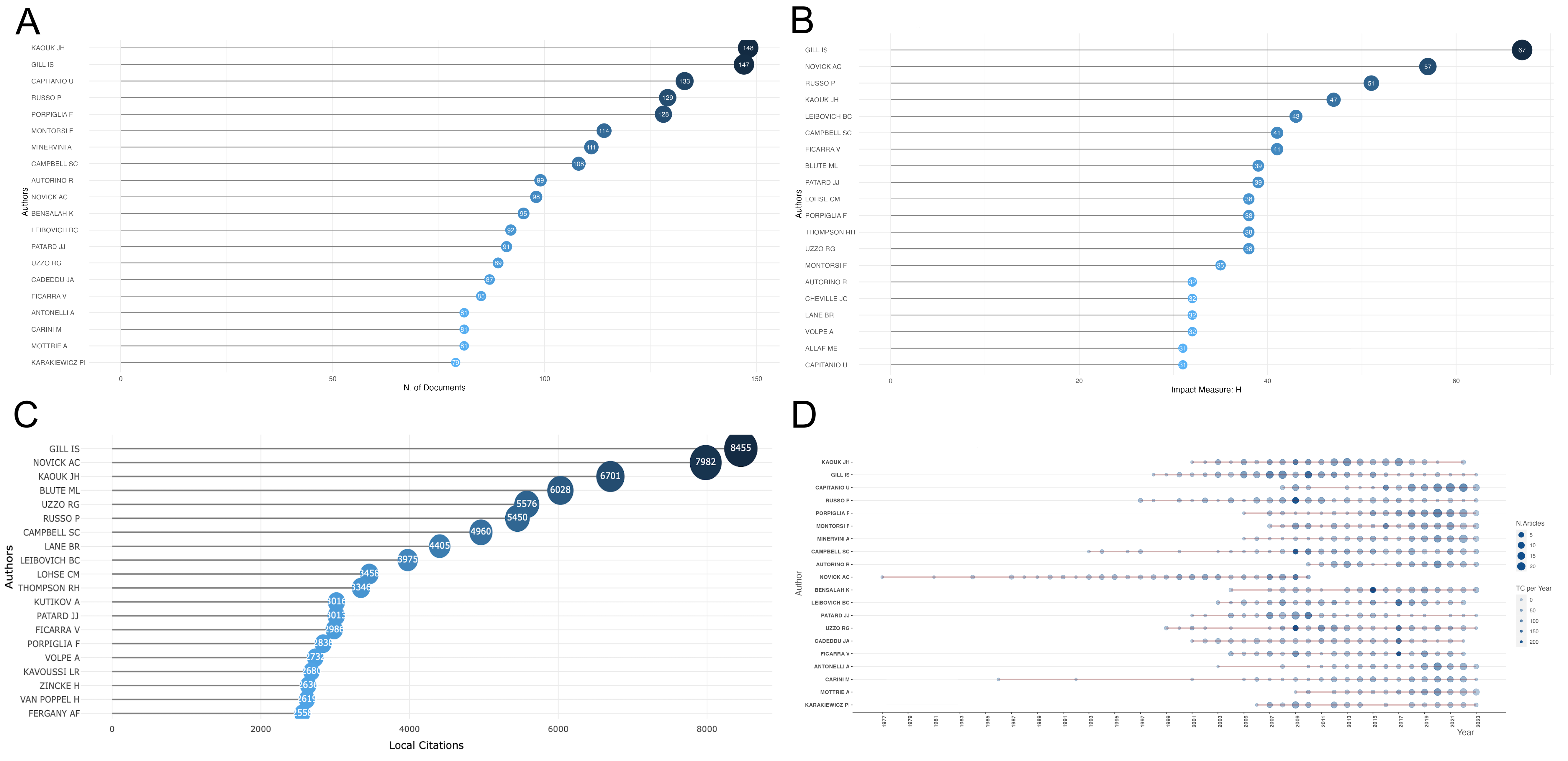
**

**Figure S3 |** Performance analysis of authors in the kidney neoplasm and nephron sparing surgery field.

**(A)** The authors who have published the most articles were regarded as the most relevant authors. The top 20 most relevant in the kidney neoplasm and nephron sparing surgery field were displayed, with “KAOUK JH” and “GILL IS” were the two most relevant authors, publishing 148 and 147 documents respectively. **(B)** The top 20 authors’ local impact ranked by h-index. It should be noted that “GILL IS” and “NOVICK AC” were the authors with the highest h-index of 67 and 57 respectively, and the h index of “KAOUK JH” ranked the fourth (47), suggesting their great influences in this field. **(C)** The top 20 most local cited authors. The most locally cited authors were also “GILL IS” and “NOVICK AC” with 8455 and 7982 local citations. Besides, other 19 authors were all cited for more than 2500 times locally. **(D)** The top 20 authors’ production over time, and the length of line means the author’s researching timeline, while the size of the nodes reflects the number of publication and the color density is proportional to total citations (TC) per year. It was noteworthy that 19 of the 20 authors were still active in the recent 2 years, again emphasizing that this was a rapidly-growing field. The two most productive authors, again “KAOUK JH” and “GILL IS” had been devoted in this area for more than twenty years, with great publications every year. TC, total citations.

| **Rank** | **Country / Region** | **Publications (%)** | **TCs** | **AACs** | **MCP (%)** |
| --- | --- | --- | --- | --- | --- |
| **1** | USA | 2801 (37.0) | 113869 | 40.7 | 327 (11.7) |
| **2** | CHINA | 765 (10.1) | 6421 | 8.4 | 30 (3.9) |
| **3** | JAPAN | 471 (6.2) | 5536 | 11.8 | 14 (3.0) |
| **4** | ITALY | 456 (6.0) | 10614 | 23.3 | 134 (29.4) |
| **5** | GERMANY | 449 (5.9) | 7800 | 17.4 | 75 (16.7) |
| **6** | FRANCE | 373 (4.9) | 6051 | 16.2 | 55 (14.7) |
| **7** | KOREA | 277 (3.7) | 3972 | 14.3 | 38 (13.7) |
| **8** | CANADA | 211 (2.8) | 7433 | 35.2 | 95 (45.0) |
| **9** | UNITED KINGDOM | 184 (2.4) | 3725 | 20.2 | 39 (21.2) |
| **10** | TURKEY | 183 (2.4) | 1363 | 7.4 | 6 (3.3) |
| **11** | SPAIN | 117 (1.5) | 654 | 5.6 | 15 (12.8) |
| **12** | AUSTRIA | 92 (1.2) | 2960 | 32.2 | 27 (29.3) |
| **13** | NETHERLANDS | 82 (1.1) | 3807 | 46.4 | 28 (34.1) |
| **14** | INDIA | 80 (1.1) | 291 | 3.6 | 4 (5.0) |
| **15** | AUSTRALIA | 76 (1.0) | 997 | 13.1 | 11 (14.5) |
| **16** | BRAZIL | 63 (0.8) | 743 | 11.8 | 11 (17.5) |
| **17** | ISRAEL | 58 (0.8) | 1221 | 21.1 | 10 (17.2) |
| **18** | POLAND | 54 (0.7) | 363 | 6.7 | 6 (11.1) |
| **19** | BELGIUM | 49 (0.6) | 2850 | 58.2 | 23 (46.9) |
| **20** | SWEDEN | 40 (0.5) | 3492 | 87.3 | 13 (32.5) |

**Table S2 |** Top 20 most contributing countries or regions in the kidney neoplasms and nephron sparing surgery field. Abbreviations: TCs, total citations; AACs, average article citations; MCP, multiple countries publication.

**
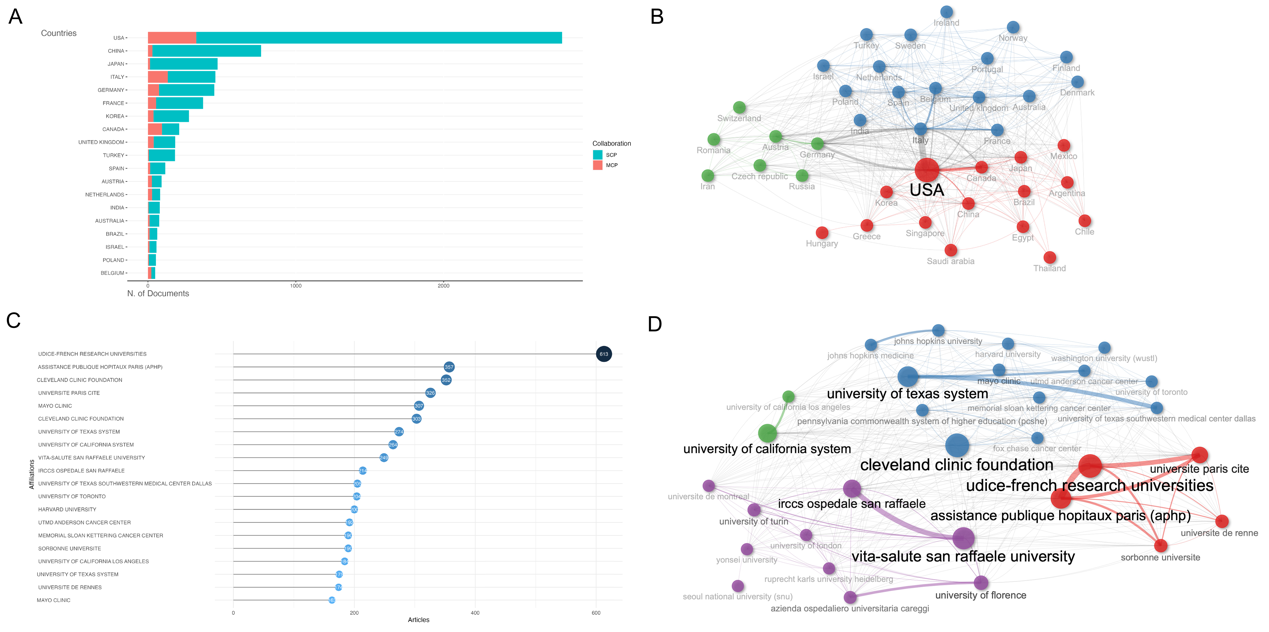
**

**Figure S4** | Performance analysis of countries and affiliations in the kidney neoplasm and nephron sparing surgery field.

**(A)** The top 20 most productive countries, ranked by the number of publications written by corresponding authors’ countries. SCP and MCP respectively means that the investigation was conducted by only one country and multiple countries. Higher MCP and higher MCP ratio suggested that the researches of the country were more likely to cooperate with others, possibly implying that the development of this field would be more advanced. We could also find that USA dominated in this area, with nearly 3000 publications. **(B)** Three clusters were identified in the country collaboration network of the top 40 productive countries. USA and Canada (red), Italy and France (blue), as well as Germany (green) were the most influential and cooperative countries in the network. **(C)** The top 20 affiliations with the most publications were shown. Overall, American and French affiliations were the two most preeminent affiliations in the field. 12 American affiliations such as CLEVELAND CLINIC FOUNDATION, MAYO CLINIC, and UNIVERSITY OF TEXAS SYSTEM, together with 5 French affiliations including UDICE-FRENCH RESEARCH UNIVERSITIES, ASSISTANCE PUBLIQUE HOPITAUX PARIS and UNIVERSITE PARIS CITE dominated in the top 20 most relevant affiliations. SCP, single country publication; MCP, multiple countries publication. **(D)** Affiliation collaboration network for kidney neoplasm and nephron sparing surgery research.

**
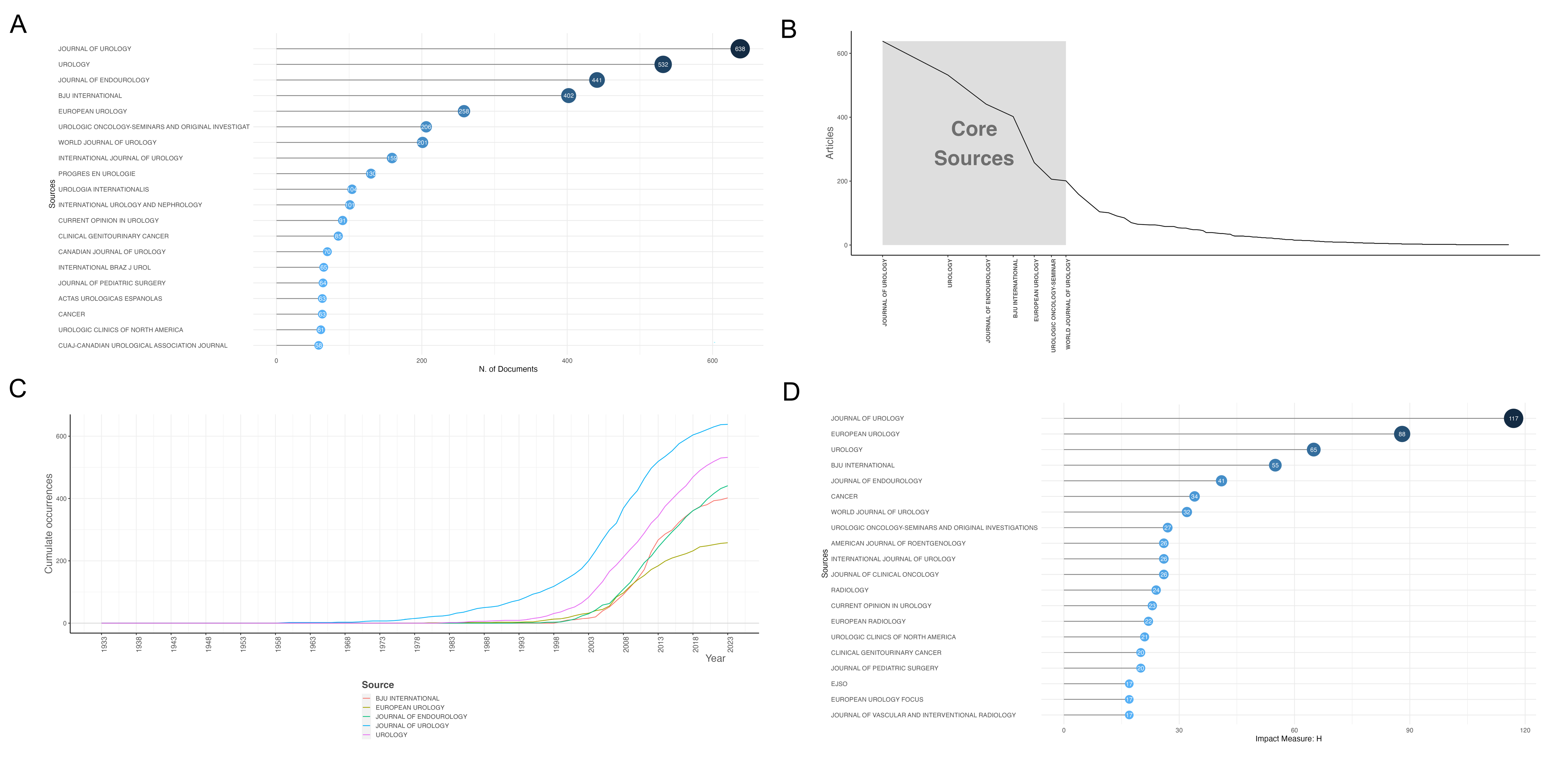
**

**Figure S5** | Performance analysis of journals in the kidney neoplasm and nephron sparing surgery field.

**(A)** The top 20 most relevant journals, measured by the number of publications. We could find that these journals were all pronounced journals in the field of urology and cancer, with a minimum of 58 publications. And journal of urology (638), urology (532), journal of endourology (441), BJU international (402), european urology (258), urologic oncology-seminars and original investigations (206), and world journal of urology (201) dominated the top seven accordingly. **(B)** With the help of the Bradford’s law, which helped us assess the exponentially diminishing returns of searching references in scientific journals, we could find that the journals in the core sources were also the previously most relevant 7 journals, further emphasizing their important role in this field. **(C)** The top five relevant journals. Among them, the number of publications of journal of urology increased steadily since 1970, and all the five journals sustained rapid growth after 2000. **(D)** The top 20 journals impact measured by h-index. The h-index of journal of urology (117), european urology (88), and urology (65) were the top three journals, revealing the publications were of high quality and had great influences on the kidney neoplasm and nephron sparing surgery field.

| **Rank** | **Journal** | **Publications** | **TCs** | **H-index** | **IF** | **QiC** |
| --- | --- | --- | --- | --- | --- | --- |
| **1** | JOURNAL OF UROLOGY | 638 | 51579 | 117 | 6.6 | Q1 |
| **2** | UROLOGY | 532 | 17464 | 65 | 2.1 | Q3 |
| **3** | JOURNAL OF ENDOUROLOGY | 441 | 8021 | 41 | 2.7 | Q3 |
| **4** | BJU INTERNATIONAL | 402 | 12496 | 55 | 4.5 | Q1 |
| **5** | EUROPEAN UROLOGY | 258 | 25754 | 88 | 23.4 | Q1 |
| **6** | UROLOGIC ONCOLOGY-SEMINARS AND ORIGINAL INVESTIGATIONS | 206 | 2785 | 27 | 2.7 | Q3 |
| **7** | WORLD JOURNAL OF UROLOGY | 201 | 3776 | 32 | 3.4 | Q2 |
| **8** | INTERNATIONAL JOURNAL OF UROLOGY | 159 | 2222 | 26 | 2.6 | Q3 |
| **9** | PROGRES EN UROLOGIE | 130 | 593 | 12 | 1.1 | Q4 |
| **10** | UROLOGIA INTERNATIONALIS | 104 | 788 | 15 | 1.6 | Q4 |
| **11** | INTERNATIONAL UROLOGY AND NEPHROLOGY | 101 | 856 | 14 | 2.0 | Q3 |
| **12** | CURRENT OPINION IN UROLOGY | 91 | 1357 | 23 | 2.5 | Q3 |
| **13** | CLINICAL GENITOURINARY CANCER | 85 | 1085 | 20 | 3.2 | Q2 |
| **14** | CANADIAN JOURNAL OF UROLOGY | 70 | 387 | 10 | 1.5 | Q4 |
| **15** | INTERNATIONAL BRAZ J UROL | 65 | 446 | 11 | 3.7 | Q2 |
| **16** | JOURNAL OF PEDIATRIC SURGERY | 64 | 1241 | 20 | 2.4 | Q2 |
| **17** | ACTAS UROLOGICAS ESPANOLAS | 63 | 196 | 7 | 1.1 | Q4 |
| **18** | CANCER | 63 | 4315 | 34 | 6.2 | Q1 |
| **19** | UROLOGIC CLINICS OF NORTH AMERICA | 61 | 2158 | 21 | 2.4 | Q3 |
| **20** | CUAJ-CANADIAN UROLOGICAL ASSOCIATION JOURNAL | 58 | 528 | 13 | 1.9 | Q4 |

**Table S3 |** The top 20 most productive journals in the renal cancer and nephron sparing surgery field. Abbreviations: TC, total citations; IF, impact factor (2023); QiC, quartile in category (2023)

**
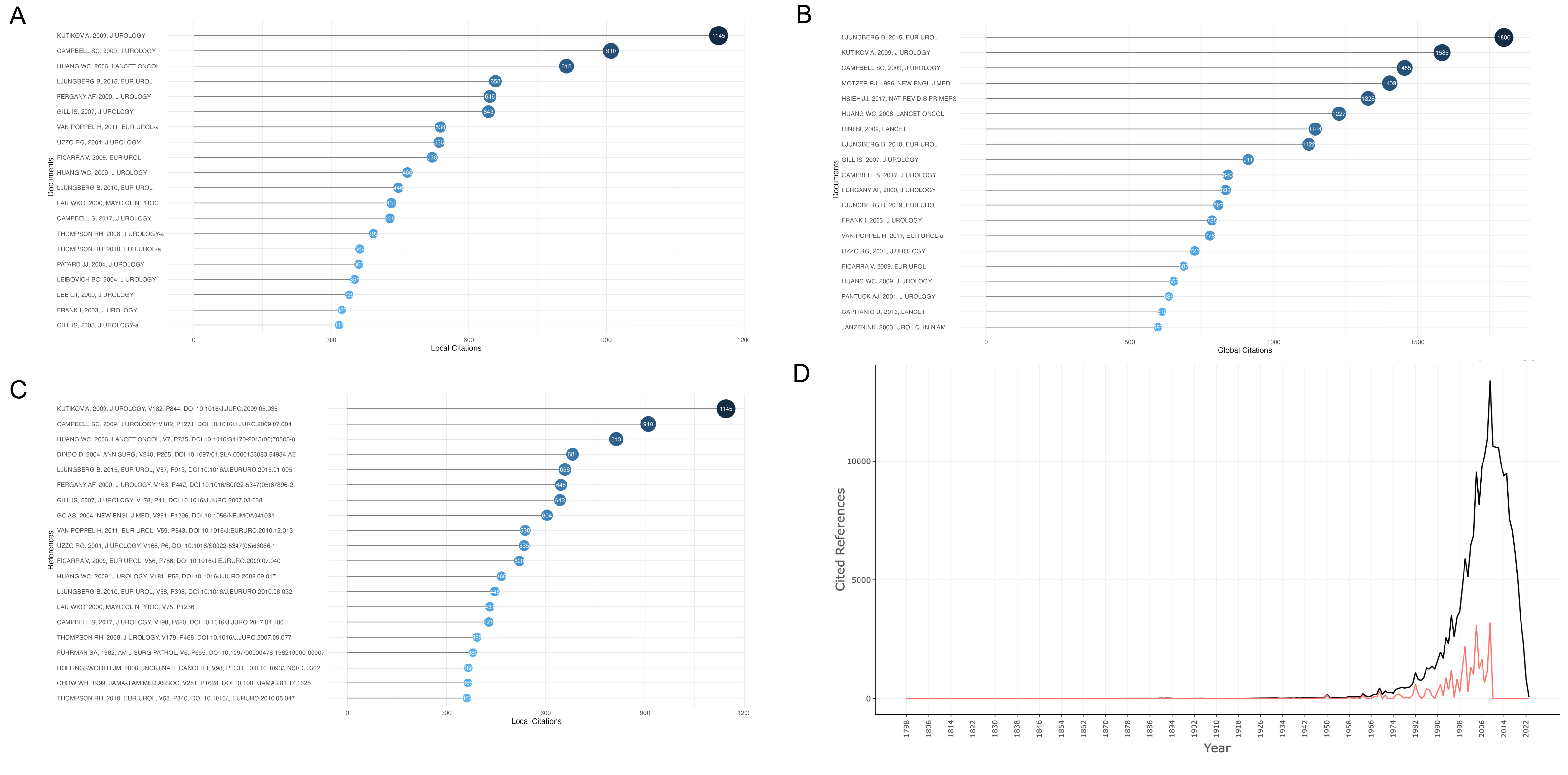
**

**Figure S6** | The most influential publications in the kidney neoplasm and nephron sparing surgery field.

**(A)** The top 20 most locally cited documents. Documents referred to the publications that we had previously downloaded in our retrieval text from the Web of science core database. Local citation meant that the document was cited by other documents in our retrieval collection. **(B)** The top 20 most totally cited documents indicating their overall great impacts. **(C)** The top 20 most locally cited references. The “references” included references of the documents we have previously retrieved, so that references with high local citations were more likely to have great influences inside this field. **(D)** References publication year spectroscopy for the kidney neoplasm and nephron sparing surgery field. Black line referred to the number of cited references, while red line meant the deviation from the 5-year median, reflecting an overall increase of cited references in this area.


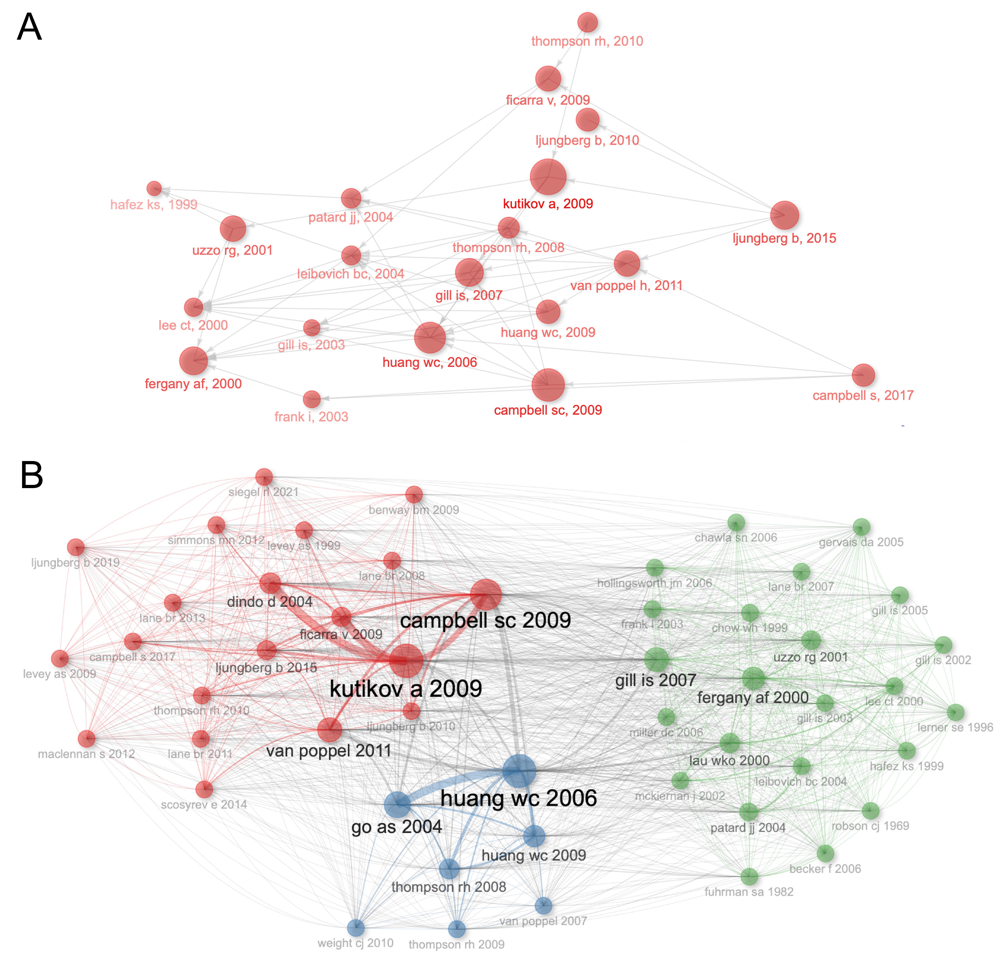


**Figure S7** | Citation analysis of the publications in the kidney neoplasm and nephron sparing surgery field.

**(A)** Historical direct citation network for kidney neoplasm and nephron sparing surgery researches. The arrows indicated the relationship of direct citation. **(B)** Co-citation network for kidney neoplasm and nephron sparing surgery researches, and three clusters were identified.

**
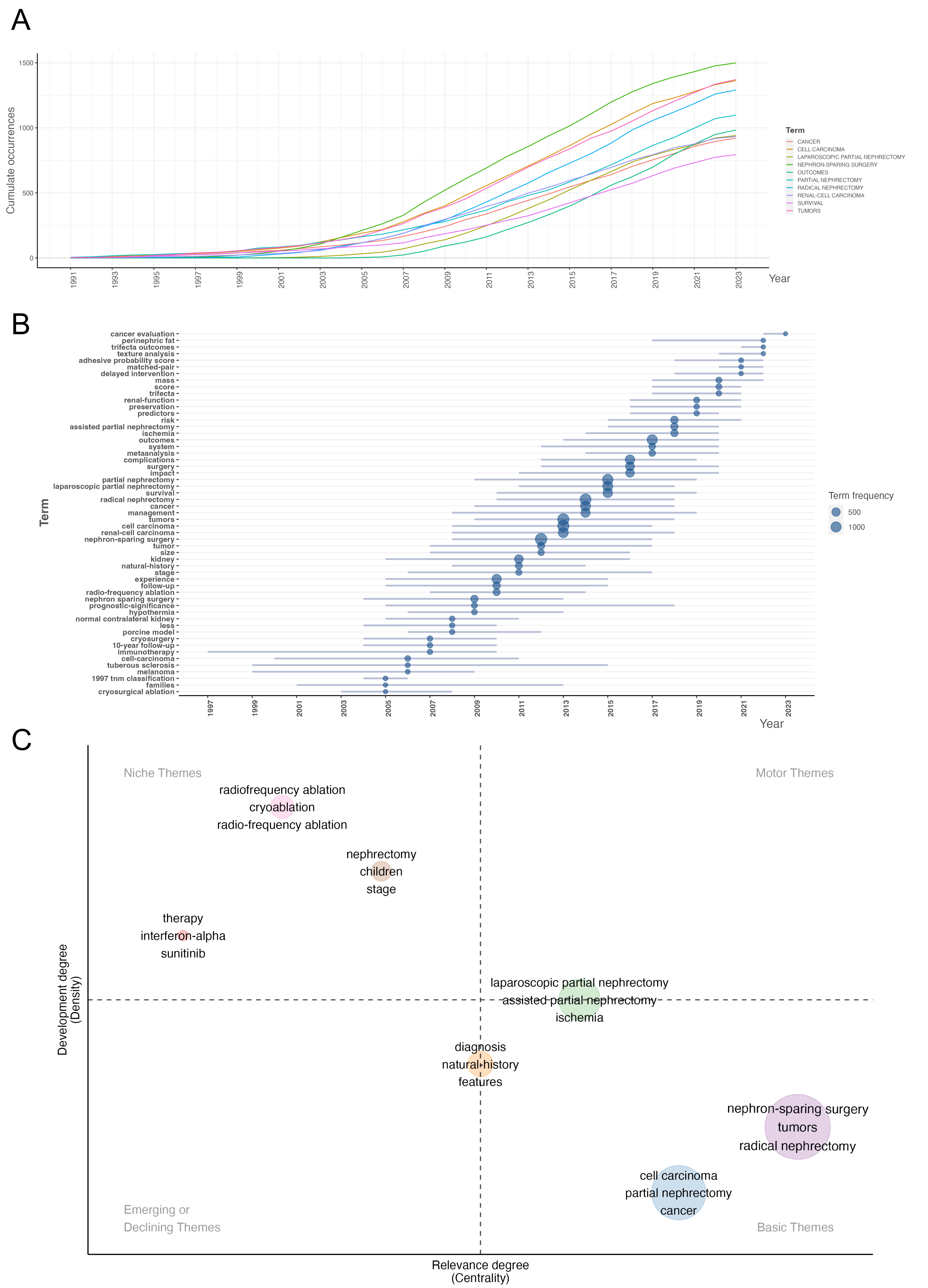
**

**Figure S8** | Performance analysis of keywords in the kidney neoplasm and nephron sparing surgery field.

**(A)** The frequency of all the top 10 most relevant words began to increase since 2005, also indicating that these keywords were the most discussed topics in recent years. **(B)** The development of trend topics over time since 1997. We could find that the surgical methods including “nephron-sparing surgery”, “radical nephrectomy”, “laparoscopic partial nephrectomy”, “assisted partial nephrectomy”, “meta-analysis” maintained the hotspots in this field for about two decades. Optimizing the surgical modalities was the core problem in this field. Other related topics like “trifecta outcomes”, “ischemia” of kidney, “complications”, “renal function”, “score” and “cancer evaluation” were all key factors in determining the outcomes of partial nephrectomy. In addition, newly emerging topics like “perinephric fat” and “texture analysis” also should be focused on. **(C)** The thematic map, which was divided into 4 quadrants (motor themes, niche themes, emerging or declining themes and basic and transversal themes) respectively. Two NSS methods including radio-frequency ablation and cryoablation, together with systemic therapies including interferon-alpha and sunitinib, were relatively more developed but less relevant to this field. While the most relevant topics further proved to be the surgical methods, as well as their influencing factors and outcomes.


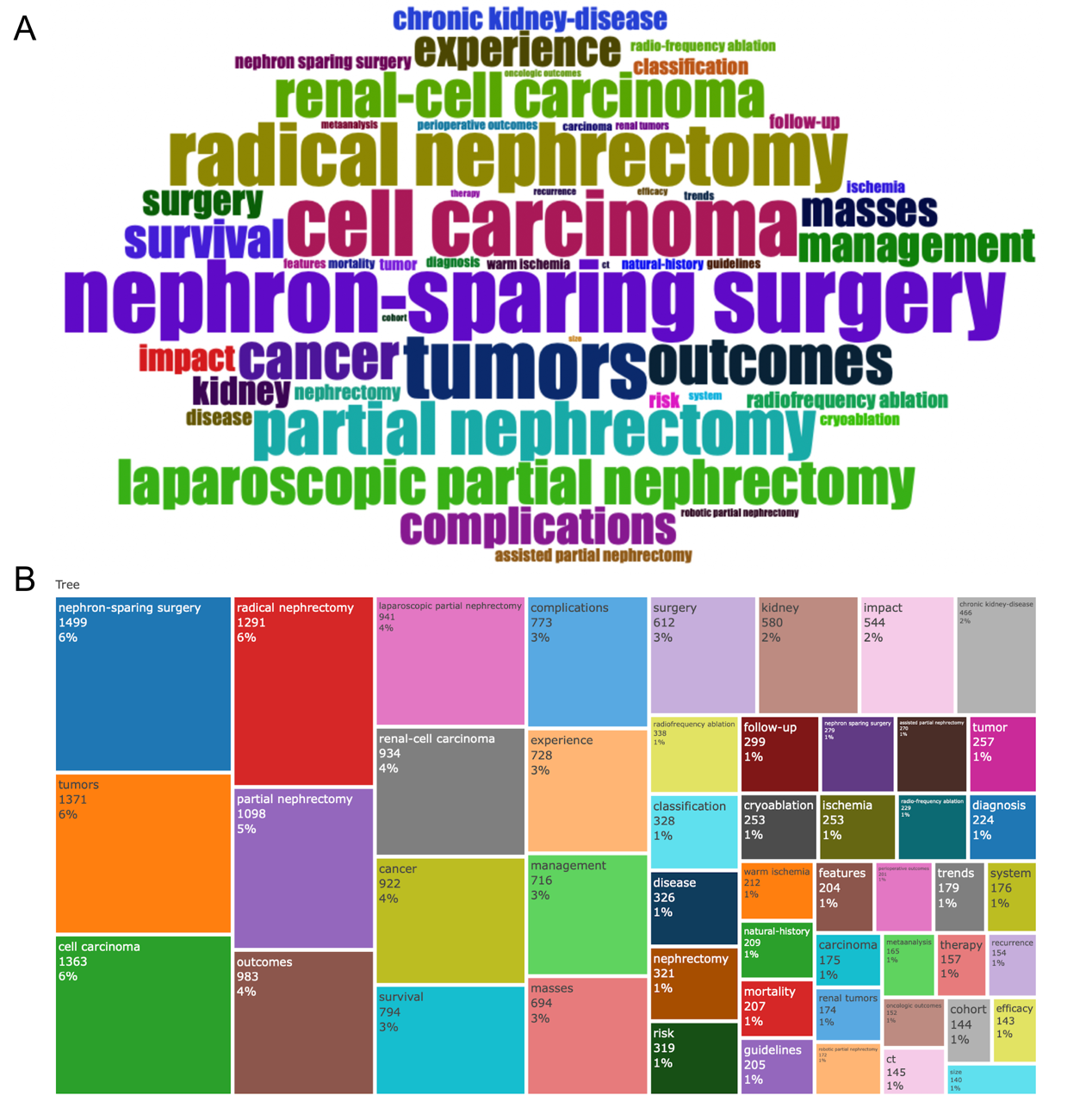


**Figure S9** | Keyword analysis of word cloud and tree map.

**(A)** Word cloud showing the top 40 most frequent words. **(B)** Tree map demonstrates the top 40 most frequent words and their frequencies in the kidney neoplasm and nephron sparing surgery field.
